# Dissection of two soybean QTL conferring partial resistance to *Phytophthora sojae* through sequence and gene expression analysis

**DOI:** 10.1186/1471-2164-13-428

**Published:** 2012-08-28

**Authors:** Hehe Wang, Asela Wijeratne, Saranga Wijeratne, Sungwoo Lee, Christopher G Taylor, Steven K St Martin, Leah McHale, Anne E Dorrance

**Affiliations:** 1 The Department of Plant Pathology, The Ohio State University, Wooster, OH 44691, USA; 2 Molecular and Cellular Imaging Center, OARDC, Wooster, OH 44691, USA; 3 Department of Horticulture and Crop Science, The Ohio State University, Columbus, OH 43210, USA

**Keywords:** QTL, Gene expression, Sequencing, SNP, Soybean, *P*. *sojae*, qRT-PCR

## Abstract

**Background:**

*Phytophthora sojae* is the primary pathogen of soybeans that are grown on poorly drained soils. Race-specific resistance to *P. sojae* in soybean is gene-for-gene, although in many areas of the US and worldwide there are populations that have adapted to the most commonly deployed resistance to *P. sojae* ( *Rps*) genes. Hence, this system has received increased attention towards identifying mechanisms and molecular markers associated with partial resistance to this pathogen. Several quantitative trait loci (QTL) have been identified in the soybean cultivar ‘Conrad’ that contributes to the expression of partial resistance to multiple *P. sojae* isolates.

**Results:**

In this study, two of the Conrad QTL on chromosome 19 were dissected through sequence and expression analysis of genes in both resistant (Conrad) and susceptible (‘Sloan’) genotypes. There were 1025 single nucleotide polymorphisms (SNPs) in 87 of 153 genes sequenced from Conrad and Sloan. There were 304 SNPs in 54 genes sequenced from Conrad compared to those from both Sloan and Williams 82, of which 11 genes had SNPs unique to Conrad. Eleven of 19 genes in these regions analyzed with qRT-PCR had significant differences in fold change of transcript abundance in response to infection with *P. sojae* in lines with QTL haplotype from the resistant parent compared to those with the susceptible parent haplotype. From these, 8 of the 11 genes had SNPs in the upstream, untranslated region, exon, intron, and/or downstream region. These 11 candidate genes encode proteins potentially involved in signal transduction, hormone-mediated pathways, plant cell structural modification, ubiquitination, and basal resistance.

**Conclusions:**

These findings may indicate a complex defense network with multiple mechanisms underlying these two soybean QTL conferring resistance to *P. sojae*. SNP markers derived from these candidate genes can contribute to fine mapping of QTL and marker assisted breeding for resistance to *P. sojae*.

## Background

*Phytophthora sojae* Kaufm. and Gerd. is a yield limiting soil borne pathogen of soybean ( *Glycine max* L. Merr.). This disease is most prevalent for soybean grown in poorly drained soils, and symptoms include pre- and post-emergence damping-off, root and stem rot, yellowing and wilting of lower leaves of the plants [1,2]. *P. sojae* is characterized as a hemi-biotrophic pathogen. *P. sojae* haustoria are produced during the early intracellular biotrophic stage and as the pathogen colonizes root tissues. At later stages of infection, light tan to brown symptoms develop leading to necrosis and cell death. Resistance to *P. sojae* in soybean is conferred by both single, dominant genes, known as *Rps* genes, that confer resistance to specific pathotypes (races) and partial resistance which is inherited as quantitative trait loci (QTL) [1,2]. In both types of resistance, zoospores move to the roots where they encyst, germinate, and penetrate within the first six hours after inoculation (hai) [3-5]. In *Rps* mediated resistance, the hyphae from avirulent *P. sojae* strains were only found in the first three cell layers. In partial resistance, hyphae colonized deeper into the cells of the root cortex. At 48 hai, hyphae were found in the same layers of root cells for soybean genotypes that are highly susceptible or had high levels of partial resistance [4]. The visible haustoria observed at 48 hai, and disease symptoms at 72 hai, suggested that the biotrophic stage of *P. sojae* occurred within the first 48 hai and the necrotrophic stage may begin approximately 72 hai in both partial resistant and susceptible soybean genotypes [4].

A total of 19 QTL have been identified in soybean genotypes resistant to *P. sojae*, of which 15 were mapped from eight separate populations from the resistant cultivar ‘Conrad’ [6-13]. Of these 15 QTLs, six mapped to chromosome (Chr.) 2 (formerly Molecular Linkage Group D1b; MLG D1b), five mapped to Chr. 13 (MLG F), two mapped to Chr. 18 (MLG G), and the remaining two mapped to Chr. 19 (MLG L) [6-8,10-12]. Interestingly, the QTL on Chr. 2 and 13 were not consistently detected with multiple isolates or the different field assays from these studies [7,8]. Individual QTL that respond differentially to specific isolates of a pathogen and environmental conditions have also been identified in several other host-pathosystems [14-17]. In order to breed for a broad-spectrum durable host resistance, the selected QTL must be able to confer resistance to multiple isolates of a pathogen, act stably under different environment conditions, explain a large percentage of the phenotypic variation (major-effect QTL), and be confirmed in different mapping populations [15]. In an earlier study, one of the QTL on Chr. 18 and two of the QTL on Chr. 19 responded similarly following inoculation to three isolates of *P. sojae* and with two different disease assay methods [11]. These three QTL also explained a significant proportion of phenotypic variation that contributed to reduced levels of root rot and lesion size. Additionally, RILs with the resistant haplotypes at these QTL had significantly higher yield than RILs with the susceptible haplotypes in field tests. These attributes make these QTL strong potential targets for breeding of broad-spectrum resistance in soybean against *P. sojae*.

**Figure 1:**
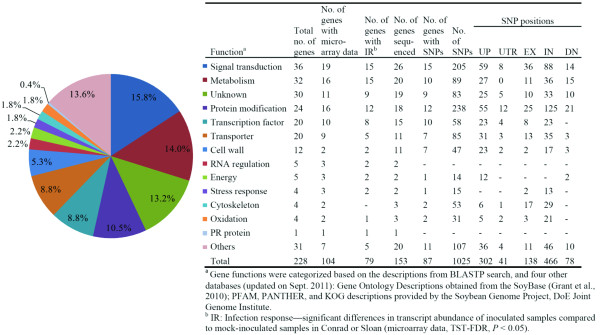
**Functional categorization of the genes underlying QTL 19–1 and 19–2 for resistance to*Phytophthora sojae***.

To improve the efficiency of incorporating these QTL into cultivar development, identifying the key genes controlling these QTL and characterizing their functions is key [15]. These genes are not only the best markers for efficient breeding, but they are also important in understanding the mechanisms that contribute to the expression of partial resistance which still remained largely unknown. In the soybean-*P. sojae* interaction, few studies have explored the molecular mechanisms that contribute to the expression of partial resistance in Conrad to *P. sojae*. Pathogenesis-related (PR) protein PR1a, PR2, basic peroxidase and matrix metalloproteinase transcript levels were reported to be higher in Conrad compared to OX 20–8 (highly susceptible) 3 days after inoculation (dai) [18]. Two studies reported that preformed suberin, a component of basal resistance, was higher in whole roots of Conrad compared to those of the susceptible line OX760-6 [5,19]. This was proposed to contribute to a 2–3 h delay in *P. sojae* penetration through the epidermis of Conrad compared to the susceptible line. Whole-genome transcription profiling of eight soybean genotypes with differential levels of partial resistance to *P. sojae*, were analyzed using soybean Affymetrix® gene chips [10,20-22]. The eight soybean genotypes were examined at 3 and 5 dai, and ~25,000 genes had statistically significant responses to infection, with little difference in transcript levels between these two sampling time points [20,22]. The infection response of four soybean genotypes, including Conrad and a susceptible cultivar Sloan, was also analyzed in a time course assay at several locations surrounding lesion development with the Affymetrix® gene chips [10,21]. Approximately 20,000 genes (53.4%) had significant changes in transcript abundance in Conrad and Sloan compared to mock inoculated controls in response to *P. sojae* infection, and the majority of changes occurred at 2, 3, and 5 dai [10]. Under the two QTL on Chr. 19, 76.0% of the genes had significant infection response in Conrad or Sloan from this microarray analysis [10] (Figure 1). Interestingly, most of the annotated functions of the genes from these regions have been reported to be involved in host defense to plant pathogens. None of the genes in this region have an *R* gene-like motif based on the Williams 82 reference genome [23].

To date, only three genes have been cloned from disease resistance QTL in plants and they each encode proteins with diverse functions [24-28], which is in accordance with the multiple hypotheses for mechanisms underlying QTL in effectively limiting pathogen colonization [16]. These three genes all had sequence variation between the resistance and susceptible alleles [25-27]. In this study, our hypotheses were that i) a complex network of defense-pathways is underlying each soybean QTL conferring resistance to *P. sojae*; ii) sequence of the genes under a QTL between resistant and susceptible genotypes are different in regions that will affect gene expression; and iii) sequence analysis will expedite the identification of potential candidate genes in soybean conferring resistance to *P. sojae*.

The two QTL on Chr. 19 responded similarly across different phenotypic assays and isolates of *P. sojae*, and a large number of defense genes associated with these QTL had significant changes in transcript abundance in response to *P. sojae* infection. Thus, they are prime candidates to explore the variation in gene sequence and expression patterns between the resistant and susceptible genotypes. Therefore, our objectives were to: i) confirm the QTL in an advanced and larger F_6:8_ Conrad × Sloan population (246 RILs); ii) examine the sequence variation of the genes underlying these QTL between Conrad and Sloan; iii) analyze the expression patterns of candidate genes representing different defense mechanisms underlying these QTL following infection by *P. sojae.* This research will not only address an expedited means to identify candidate genes in soybean conferring resistance to *P. sojae*, but also provide more polymorphic markers for further fine mapping of the QTL regions.

## Results and discussion

### QTL confirmation in F_6:8_ population

**Figure 2:**
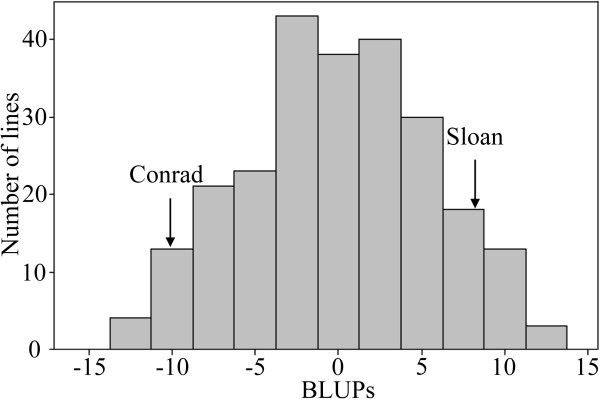
**Lesion distribution of the F**_**6:8**_**‘Conrad × Sloan’ population inoculated with*Phytophthora sojae***.

These two QTL were identified previously in the F_4:6_ Conrad × Sloan population of 186 RILs [10,11]. In tray test assays carried out in the present study, best linear unbiased predictor (BLUP) values [29] from root lesion lengths measured at 7 dai from *P. sojae* isolate 1.S.1.1 in the Conrad × Sloan F_6:8_ RIL population ranged from −12.9 to 12.7 (lesion length 21.5 to 51.9 mm) with a normal distribution, indicating that the resistance was quantitatively inherited (Figure 2). The broad-sense heritability estimate for lesion length was 0.87. Conrad has high levels of partial resistance to *P. sojae* and Sloan is moderately susceptible. Both of cultivars performed consistently as checks across replicates. Hereafter, Conrad and Sloan will be referred to as the R and S genotypes, respectively. Five QTL with resistance alleles from R cultivar, two each on Chr. 18 and 19 and one on Chr. 1 (MLG D1A), were identified, each explaining 6.0-19.6% of the phenotypic variation for a total of 67.2% for interval mapping (IM), and 4.8-11.9% of the phenotypic variation for a total of 37.1% for composite interval mapping (CIM) (Table 1).

**Table 1:** **QTL from Conrad against*Phytophthora sojae*mapped using the F**_**6:8**_**Conrad × Sloan population**

**QTL**^**a**^	**IM**		**CIM**		**Marker**^**b**^	**Selected lines for qRT-PCR**^**c**^					
	**LOD**	**Exp. Var. (%)**	**LOD**	**Exp. Var. (%)**		**R**	**2022**	**1960**	**1974**	**1854**	**S**
19-1	6.0	11.8	3.5	4.8	BARC-047496-12943	+	+	+	-	-	-
					Satt527	+	+	+	-	-	-
					Glyma19g35340	+	+	+	-	-	-
					GML_OSU10^d^	+	+	+	-	-	-
					BARCSOYSSR_19_1243	+	+	+	-	-	-
19-2	9.4	18.1	7.9	11.9	Glyma19g40800	+	+	+	-	-	-
					BARCSOYSSR_19_1452^d^	+	+	+	-	-	-
					Glyma19g40940	+	+	+	-	-	-
					Glyma19g41210	+	+	+	-	-	-
					Glyma19g41390	+	+	+	-	-	-
					BARCSOYSSR_19_1473	+	+	+	-	-	-
					Glyma19g41580	+	+	+	-	-	-
					Glyma19g41780	+	+	+	-	-	-
					Glyma19g41800	+	+	+	-	-	-
					Glyma19g41870	+	+	+	-	-	-
					Glyma19g41900	+	+	+	-	-	-
					Glyma19g42120	+	+	+	-	-	-
					Glyma19g42200	+	+	+	-	-	-
					Glyma19g42220	+	+	+	-	-	-
					Glyma19g42240	+	+	+	-	-	-
					Glyma19g42340	+	+	+	-	-	-
					Glyma19g42390	+	+	+	-	-	-
					BARC-039977-07624	+	+	+	-	-	-
1	3.1	6.0	3.7	5.0	BARC-060037-16311	+	+	-	+	-	-
					BARC-064441-18673^d^	+	+	-	+	-	-
					BARC-054071-12319	+	+	-	+	-	-
18-1	5.3	11.7	4.5	6.1	BARCSOYSSR_18_1707^d^	+	-	-	+	+	-
					BARCSOYSSR_18_1710	+	-	-	+	+	-
18-2	10.5	19.6	8.4	9.3	BARCSOYSSR_18_1777	+	-	-	+	+	-
					BARC-057845-14952	+	-	-	+	+	-
					BARC-039397-07314^d^	+	-	-	+	+	-
					BARCSOYSSR_18_1949	+	-	-	+	+	-

The QTL presence in the six selected lines for qRT-PCR is also shown.

^a^ QTL were presented as chromosome number, followed by the serial number if there were more than one on the same chromosome;

^b^ Markers tested in each QTL interval: markers started with ‘BARC’ or ‘Sat’ are from the public databases (Choi et al., 2007; Hyten et al., 2010; Song et al., 2010), and markers started with ‘Glyma’ are the PAMSA markers designed in this study;

^c^ Conrad (R) alleles for each marker were presented as “+” in the selected lines for qRT-PCR, while “-” referred to the Sloan (S) alleles;

^d^ Nearest marker under each QTL interval.

The QTL 18–2, 19–1, and 19–2, which confer resistance to multiple *P. sojae* isolates, were first mapped in a Conrad × Sloan F_4:6_ population using two different phenotypic methods [10,11]. In this study, all three QTL were confirmed in the larger F_6:8_ generation, flanked by similar markers as in the F_4:6_ population. The QTL 18–2 (Gm18: 59016134 to 62263273) co-localized with the position of the *R*-gene mediated resistance to *P. sojaeRps4* (flanked by markers BARC-031121-06998 and BARC-031193-07008, Gm18: 60469824 to 60780954) and *Rps6* (flanked by markers Sat_372 and BARC-017669-03102, Gm18: 61095646 to 62046327) [30-32]. Residual function of defeated *R*-genes has been proposed to contribute to the expression of partial resistance in other host-pathogen systems [16]. However, the R genotype in this study does not have known *Rps* genes, nor does this locus have isolate specificity to *P. sojae*. Direct sequencing of this QTL region would be necessary to assess if *R*-gene-like sequences were present in R or S genotypes. In contrast to QTL 18–2, there were no *R* gene-like sequences in Williams 82 where the QTL 19–1 and 19–2 mapped, which indicates the mechanisms underlying these two QTL that contribute to the expression of partial resistance are likely to be different than *R*-gene mediated resistance. The QTL 19–1 and 19–2 spanned ~4.0 cM and ~4.8 cM, respectively, on the soybean consensus map v4.0 [33]. In this study, these two QTL had the log of odds likelihood LOD scores of 3.5 and 7.9 (CIM), and accounted for 4.8 and 11.9% of the variation in lesion length, respectively (Table 1). The expression of broad-spectrum resistance to multiple isolates, consistent detection through different phenotypic assays, and detection in two generations of the same population, make the two QTL on Chr. 19 prime targets to examine the genetic and mechanistic contributions towards the expression of partial resistance to *P. sojae* in soybean. These QTL regions are large and encompass many genes, thus, numerous molecular markers per locus may be required to ensure successful introgression of the critical component(s) of the locus for full expression of resistance in cultivars.

### Sequence variation of genes underlying the QTL between R and S cultivars

**Figure 3:**
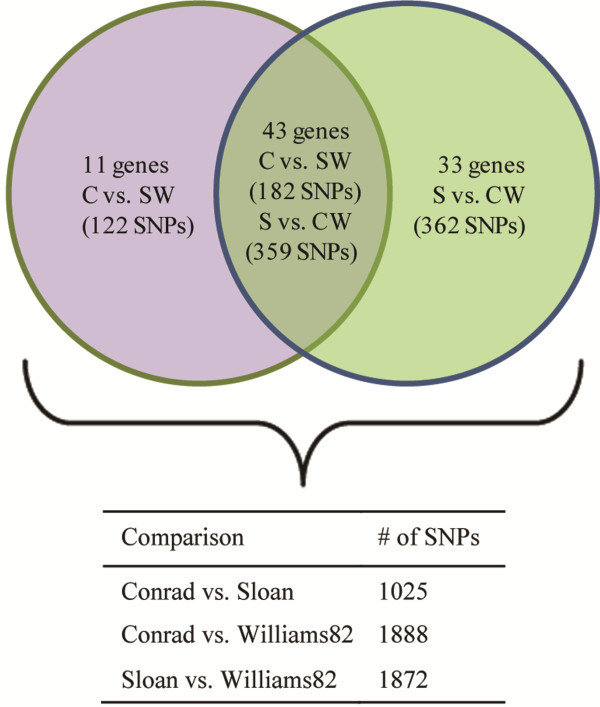
**SNPs detected between Conrad (C), Sloan (S), and Williams 82 (W).** The 1025 SNPs between Conrad and Sloan were located in 87 genes, which were shown on the graph as three groups, and the number of genes in each group had only SNPs detected in the listed comparison.

The QTL 19–1 and 19–2 spanned ~0.5 Mb (Gm19: 42819782 to 43332226) and 1.5 Mb (Gm19: 47108989 to 48606553), respectively, on the physical map [23]. A total of 53 and 175 genes were within QTL 19–1 and 19–2, respectively (Additional files 1 and 2). These genes were classified into 14 functional categories (Figure 1). Of these 228 genes, 11 from QTL 19–1 and 142 from QTL 19–2 were successfully amplified in R and S genotypes with LR-PCR and sequenced with Illumina GA II, including 1.2 kb upstream and 400 bp downstream of the gene coding regions. A total of 1025 single nucleotide polymorphisms (SNPs) were identified between R and S in 87 genes (nine genes from QTL 19–1 and 76 from QTL 19–2) (Figures 1 and 3, Additional file 3). The ‘Transcription factor’ (10 of the 15 genes) and ‘Protein modification’ (12 of the 18 genes) functional groups had the highest percentage of genes with SNPs between R and S (Figure 1). Of the 79 genes with significant infection response in R or S from the previous microarray studies [10,21], 53 were successfully sequenced and 414 SNPs were identified from 29 genes (55% of sequenced genes). For comparison, 17 of the 25 genes with no infection response from the microarray results were sequenced and 154 SNPs were identified from 10 genes (59% of sequenced genes).

Among these sequences, there was a greater number of SNPs in the introns and 1.2 kb upstream regions compared to the exons and UTRs (Figure 1). This was expected and similar to that observed in the soybean genome reported from other studies [34]. There was an average of 1.6 single nucleotide polymorphisms (SNPs) per 1000 bp (0.16%), which was slightly higher than the average SNP frequency (1 SNP per 1000 bp) in the genic and perigenic regions of soybean cultivars calculated from previous studies [35,36]. Marker assays using the 1,536 SNPs from the “Universal Soy Linkage Panel”, estimated an average of 458 SNPs for each pair of soybean cultivars based on pair wise comparisons of 96 elite cultivars [33]. Only 320 of the 1536 SNPs were polymorphic between the R and S genotypes, which indicated that the polymorphism between R and S was slightly lower than the average for pairs of soybean cultivars [Cregan and Dorrance, unpublished data]. Thus, an elevated SNP frequency in the genic and perigenic regions of defense-related genes may reflect the sequence differences in these regions that control the phenotypic differences in resistance between R and S.

Of the total 1025 SNPs between R and S, 304 SNPs located in 54 genes within QTL 19–2 occurred in R compared to both S and Williams 82 (Figure 3). There were 11 genes that had 122 SNPs unique in R when compared to S and Williams 82 (Figure 3, Table 2). Both S and Williams 82 have lower levels of partial resistance than R, hence we hypothesized that these 304 SNPs, especially those in the 11 genes, are more likely to contribute to the expression of high levels of partial resistance. Of these 304 SNPs, 21 were non-synonymous, located in eight genes (Table 3) and potentially contribute to the differences in partial resistance by modified protein structure(s).

**Table 2:** Eleven genes in which Conrad had unique SNPs vs. Sloan & Williams82

**GlymaID/Affy ID**^**a**^	**PFAM**^**b**^	**GO function**^**c**^	**PANTHER**^**d**^	**KOG**^**e**^	**BLASTP**	**E-value**	**BLAST hit species**	**# of SNPs**	**SNP locations**	**IR**^**f**^**_C**	**IR_S**
Glyma19g40800/-	WD domain, G-beta repeat	-	WD repeat protein	WD-repeat protein WDR6, WD repeat superfamily	Transducin/WD40 domain-containing protein	0E+00	*Arabidopsis thaliana*	47	exon, intron, downstream		
Glyma19g40840/-	Pectinesterase/Plant invertase/pectin methylesterase inhibitor	Pectinesterase activity; cell wall; cell wall modification	-	-	Pectinesterase; Pectinesterase inhibitor	0E+00	*Medicago truncatula*	1	downstream		
Glyma19g40940/-	Glycosyl hydrolases family 28	Carbohydrate metabolism; polygalacturonase activity	-	-	Glycoside hydrolase family 28 protein	0E+00	*Arabidopsis thaliana*	2	upstream, intron		
Glyma19g41590/ Gma.14131.1.S1_atGmaAffx.26456.1.S1_at GmaAffx.76884.1.S1_at	Haloacid dehalogenase-like hydrolase|Redoxin|NHL repeat	Hydrolase activity	2-deoxyglucose-6-phosphate phosphatase 2	Predicted haloacid-halidohydrolase and related hydrolases	2-deoxyglucose-6-phosphate phosphatase, putative	0E+00	*Ricinus communis*	1	intron	-	-
Glyma19g41900/-	F-box domain	-	-	-	Phloem-specific lectin PP2-like protein	2E-82	*Arabidopsis thaliana*	8	upstream, 5' UTR, exon, downstream		
Glyma19g42120/ Gma.14232.1.S1_at	-	-	-	Uncharacterized conserved protein	heparan-alpha-glucosaminide N-acetyltransferase	0+00	*Arabidopsis thaliana*	1	intron	−2,-3	−2,-3,-5
Glyma19g42200/ Gma.9498.1.S1_a_at	Rapid ALkalinization Factor (RALF)	Signal transducer activity	-	-	Rapid ALkalinization Factor	9e-47	Medicago truncatula	11	upstream, intron	2	-
Glyma19g42210/ GmaAffx.69813.1.A1_at	RAD9	DNA repair	DNA repair protein RAD9	Checkpoint 9-1-1 complex, RAD9 component	Rad9	0E+00	*Medicago truncatula*	12	upstream, exon, intron	-	-
Glyma19g42220/ GmaAffx.33386.1.A1_at	Respiratory burst NADPH oxidase; EF hand; Ferric reductase like transmembrane component; FAD-binding domain; Ferric reductase NAD binding domain	Calcium ion binding iron ion binding oxidoreductase activity; FAD binding	NADPH oxidase	Ferric reductase, NADH/NADPH oxidase and related proteins	Respiratory burst oxidase 2	0E+00	*Medicago truncatula*	29	upstream, exon, intron	2	2
Glyma19g42240/ Gma.13144.1.S1_at	Core histone H2A/H2B/H3/H4;Histone-like transcription factor (CBF/NF-Y) and archaeal histone	DNA binding	Histone H2A	Histone 2A	Histone H2A 7	2e-73	*Arabidopsis thaliana*	2	upstream, 5' UTR	−2,5	3,5
Glyma19g42390/-	Cyclin, N-terminal domain	-	Family not named	Cyclin	Cyclin-dependent protein kinase, putative	6e-59	*Ricinus communis*	8	upstream, intron, downstream		

^a^ GlymaID/Affymetrix probe IDs that match with the predicted genes underlying QTL (http://soybase.org/AffyChip/), where “-” means there was no Affymetrix IDs available for the specific gene;

^b^ PFAM description provided by the Soybean Genome Project, DoE Joint Genome Institute (http://www.phytozome.net/soybean.php, updated on July 2011);

^c^ Gene Ontology Descriptions obtained from the (http://soybase.org, updated on July 2011);

^d^ PANTHER description provided by the Soybean Genome Project, DoE Joint Genome Institute (http://www.phytozome.net/soybean.php, updated on July 2011);

^e^ KOG Description assigned by the Soybean Genome Project, DoE Joint Genome Institute (http://www.phytozome.net/soybean.php, updated on July 2011);

^f^ IR: Infection response—significant differences in transcript abundance of inoculated samples compared to mock-inoculated samples in Conrad (C) or Sloan (S) (microarray data, TST-FDR, *P* < 0.05) at specified time points (dai); where “-” means there was no significant response observed at any time point after inoculation, a positive value indicates that the gene was up-regulated at the specified time point, and a negative value indicates the gene was down-regulated at the specified time point [10,21].

**Table 3:** SNPs causing non-synonymous changes in Conrad (C) genes compared to Sloan (S) and Williams82 (W)

**GlymaID/Affy ID**^**a**^	**PFAM**^**b**^	**GO function**^**c**^	**PANTHER**^**d**^	**KOG**^**e**^	**BLASTP function**	**E-value**	**BLAST hit species**	**SNP position**^**f**^	**Amino acid**		**IR**^**g**^	
									**C**	**SW**	**C**	**S**
Glyma19g40800/	WD domain, G-beta repeat	-	WD repeat protein	WD-repeat protein WDR6, WD repeat superfamily	Transducin/WD40 domain-containing protein	0E+00	*Arabidopsis thaliana*	47113947	V	M		
								47116418	L	V		
								47116517	T	A		
								47116586	I	V		
								47116637	G	R		
								47118102	G	D		
								47118156	N	S		
								47118234	S	L		
								47118485	Y	H		
								47119034	V	I		
Glyma19g41230/ -	POT family	Oligopeptide transport; membrane; transporter activity	Oligopeptide transporter-related	H+/oligopeptide symporter	Nitrate transporter, putative	0E+00	*Ricinus communi*	47535046	Q	K		
Glyma19g41630/ GmaAffx.82770.1.S1_at	Nicotianamine synthase protein	Nicotianamine synthase activity	-	-	Nicotianamine synthase	2E-177	*Lotus japonicus*	47867334	R	K	−3,-5	−3,-5
Glyma19g41740/-	Calmodulin binding protein-like	Calmodulin binding	-	-	Calmodulin-binding protein, putative	2E-50	*Oryza sativa*	47939823	S	A		
								47940855	L	M		
Glyma19g41800/GmaAffx.67321.1.S1_at	Kinesin motor domain	ATPase activity microtubule binding microtubule motor activity	Kinesin heavy chain	Kinesin (KAR3 subfamily)	Kinesin heavy chain, putative	0E+00	*Ricinus communis*	47974179	M	K	-	-
								47974243	N	K		
								47974369	STOP	Y		
Glyma19g41900/-	F-box domain	-	-	-	Phloem-specific lectin PP2-like protein	2E-82	*Arabidopsis thaliana*	48050493	P	S		
Glyma19g42210/ GmaAffx.69813.1.A1_at	RAD9	DNA repair	DNA repair protein RAD9	Checkpoint 9-1-1 complex, RAD9 component	Rad9	0E+00	*Medicago truncatula*	48232086	A	V	-	-
Glyma19g42220/ GmaAffx.33386.1.A1_at	Respiratory burst NADPH oxidase|EF hand|Ferric reductase like transmembrane component|FAD-binding domain|Ferric reductase NAD binding domain	Calcium ion binding iron ion binding oxidoreductase activity FAD binding	NADPH oxidase	Ferric reductase, NADH/NADPH oxidase and related proteins	Respiratory burst oxidase 2	0E+00	*Medicago truncatula*	48239628	G	A	2	2
								48240340	F	S		

^a^ GlymaID/Affymetrix probe IDs that match with the predicted genes underlying QTL (http://soybase.org/AffyChip/), where “-” means there was no Affymetrix IDs available for the specific gene;

^b^ PFAM description provided by the Soybean Genome Project, DoE Joint Genome Institute (http://www.phytozome.net/soybean.php, updated on July 2011);

^c^ Gene Ontology Descriptions obtained from the (http://soybase.org, updated on July 2011);

^d^ PANTHER description provided by the Soybean Genome Project, DoE Joint Genome Institute (http://www.phytozome.net/soybean.php, updated on July 2011);

^e^ KOG Description assigned by the Soybean Genome Project, DoE Joint Genome Institute (http://www.phytozome.net/soybean.php, updated on July 2011);

^f^ SNP position shown as their coordinate on Chr. 19 reference sequence (http://www.phytozome.net/soybean.php, Glyma1);

^g^ IR: Infection response—significant differences in transcript abundance of inoculated samples compared to mock-inoculated samples in Conrad (C) or Sloan (S) (microarray data, TST-FDR, *P* < 0.05) at specified time points (dai); where “-” means there was no significant response observed at any time point after inoculation, a positive value indicates that the gene was up-regulated at the specified time point, and a negative value indicates the gene was down-regulated at the specified time point [10,21].

Twenty-seven of 29 selected SNPs (one per gene) were verified by a modified polymerase chain reaction (PCR) amplification of multiple specific alleles (PAMSA) [37]. Locus-specific primers could not be found for the SNP in Glyma19g42510, of which a highly homologous copy was present on Chr. 3. Sanger sequencing of the PAMSA amplicons of the second gene not confirmed by PAMSA, Glyma19g41420, identified a 22 bp deletion instead of the predicted SNPs at 918–939 bp upstream (Additional file 4). This gene is predicted to encode a serine/threonine protein kinase and microarray analysis indicated a similar level of down-regulation in both R and S at 2 dai [10].Thus, the deletion in the upstream region most likely did not cause the infection response observed in both R and S in the microarray analysis.

### Candidate genes underlying the QTL and their expression patterns

Microarray data was available for 21 of the 53 genes from QTL 19–1, and 83 of the 175 genes under QTL 19–2 [10,21]. Of these, 15 genes from QTL 19–1 and 64 from QTL 19–2 responded significantly to infection in R or S genotypes. The highest percentages of genes (78.9-93.8%) with infection response were observed in the ‘Signal transduction’, ‘Metabolism’, ‘Unknown’, and ‘Transcription factor’ categories (Figure 1). To further differentiate the potential candidate defense genes within the QTL regions, one gene from QTL 19–1 and 18 genes from QTL 19–2 were examined for their expression patterns in response to *P. sojae* infection at 12, 24, 48, and 72 hai in R, S, and four selected RILs using qRT-PCR (Tables 1,2,3,4, and 5, Additional files 1, and 2). The genes were selected based on their annotated functions, sequence variation and differential expression patterns from microarray data between R and S. Eight of the genes had microarray data with significant infection response in R, of which six genes had SNPs between R and S. A total of 15 genes in qRT-PCR assay had SNPs between R and S, with eight genes harboring unique sequence in R compared to both S and Williams 82. The presence of R and S alleles of these genes in the four RILs was verified by PAMSA (Table 1).

**Table 4:** Genes with significant expression differences in Conrad vs. Sloan in qRT-PCR

**GlymaID**	**PFAM**^**a**^	**GO function**^**b**^	**PANTHER**^**c**^	**KOG**^**d**^	**BLASTP**	**E-value**	**BLAST hit species**	**Contrasts**	**Sampling points (hai)**				
									**12**	**24**	**48**	**72_I**^**h**^	**72_U**^**i**^
Glyma19g35340	Zinc-binding dehydrogenase	Zinc ion binding	Alcohol dehydrogenase related	Alcohol dehydrogenase, class III	Alcohol dehydrogenase, putative	0E + 00	*Ricinus communis*	CC^e^				−1.8	
								RC^f^				2.0	
								IC^g^			−1.5		
Glyma19g40800	WD domain, G-beta repeat	-	WD repeat protein	WD-repeat protein WDR6, WD repeat superfamily	Transducin/WD40 domain-containing protein	0E + 00	*Arabidopsis thaliana*	CC				−2.4	
								RC				2.4	
								IC					−1.9
Glyma19g40940	Glycosyl hydrolases family 28	Carbohydrate metabolism; polygalacturonase activity	-	-	Glycoside hydrolase family 28 protein	0E + 00	*Arabidopsis thaliana*	CC					1.7
								RC			−1.9	−1.6	
								IC			−2.2		
Glyma19g40950	WRKY DNA -binding domain	Transcription factor activity; sequence-specific DNA binding	-	-	Putative WRKY transcription factor 42	9E-91	*Arabidopsis thaliana*	CC				−1.7	
								RC				2.0	
								IC					
Glyma19g40970	AUX/IAA family	Transcription factor activity	-	-	Auxin-responsive protein IAA20, putative	1E-48	*Ricinus communis*	CC					
								RC					−2.1
								IC					−2.4
Glyma19g41580	-	-	-	-	Transcription factor bHLH149	1E-30	*Arabidopsis thaliana*	CC			−2.7	−7.3	−3.2
								RC			1.5	7.3	2.5
								IC			−1.9		
Glyma19g41800	Kinesin motor domain	ATPase activity microtubule binding microtubule motor activity	Kinesin heavy chain	Kinesin (KAR3 subfamily)	Kinesin heavy chain, putative	0E + 00	*Ricinus communis*	CC	−7.0	−6.6	−9.1	−6.4	−3.4
								RC				2.0	
								IC	−5.4	−4.8	−8.6	−3.2	−4.7
Glyma19g41900	F-box domain	-	-	-	Phloem-specific lectin PP2-like protein	2E-82	*Arabidopsis thaliana*	CC				−1.9	−1.9
								RC			2.0		1.5
								IC	1.8				
Glyma19g41930	Leucine Rich Repeat	Protein binding	F-box/leucine rich repeat protein	Leucine rich repeat proteins, some proteins contain F-box	Ubiquitin-protein ligase, putative	0E + 00	*Ricinus communis*	CC					
								RC				3.3	
								IC					
Glyma19g42050	Calcineurin-like phosphoesterase	Protein serine/threonine phosphatase activity	Serine/threonine protein phosphatase	Serine/threonine specific protein phosphatase PP1, catalytic subunit	Serine/threonine-protein phosphatase PP1 isozyme 8	0E + 00	*Arabidopsis thaliana*	CC					
								RC			1.7		
								IC					
Glyma19g42120	-	-	-	Uncharacterized conserved protein	heparan-alpha-glucosaminide N-acetyltransferase	0 + 00	*Arabidopsis thaliana*	CC				−2.1	
								RC				3.6	2.4
								IC					
Glyma19g42200	Rapid ALkalinization Factor (RALF)	Signal transducer activity	-	-	Rapid ALkalinization Factor	9e-47	*Medicago truncatula*	CC					
								RC			1.8		−3.9
								IC	1.5				−3.4
Glyma19g42220	Respiratory burst NADPH oxidase; EF hand; Ferric reductase like transmembrane component; FAD-binding domain; Ferric reductase NAD binding domain	Calcium ion binding iron ion binding oxidoreductase activity; FAD binding	NADPH oxidase	Ferric reductase, NADH/NADPH oxidase and related proteins	Respiratory burst oxidase 2	0E + 00	*Medicago truncatula*	CC					
								RC					
								IC	1.8				
Glyma19g42240	Core histone H2A/H2B/H3/H4;Histone-like transcription factor (CBF/NF-Y) and archaeal histone	DNA binding	Histone H2A	Histone 2A	Histone H2A 7	2e-73	*Arabidopsis thaliana*	CC					
								RC					−1.9
								IC					
Glyma19g42340	Protein tyrosine kinase	Protein-tyrosine kinase activity; protein amino acid phosphorylation; ATP binding	Mapkk-related serine/threonine protein kinases	MEKK and related serine/threonine protein kinases	NPK1-related protein kinase 1 L	0E + 00	*Arabidopsis thaliana*	CC					
								RC			2.4	2.9	
								IC				2.5	
Glyma19g42460	Core histone H2A/H2B/H3/H4|Histone-like transcription factor (CBF/NF-Y) and archaeal histone	DNA binding transcription factor activity	Histone-like transcription factor ccaat-related	CCAAT-binding factor, subunit C (HAP5)	ccaat-binding transcription factor, putative	1e-114	*Ricinus communis*	CC					
								RC				1.5	
								IC					

*P* < 0.05, Fold difference > 1.5. Empty cell indicates that there was no significant difference, and a negative value indicates the fold that Sloan’s expression ratio was greater than Conrad’s.

^a^ PFAM description provided by the Soybean Genome Project, DoE Joint Genome Institute (http://www.phytozome.net/soybean.php, updated on July 2011);

^b^ Gene Ontology Descriptions obtained from the (http://soybase.org, updated on July 2011);

^c^ PANTHER description provided by the Soybean Genome Project, DoE Joint Genome Institute (http://www.phytozome.net/soybean.php, updated on July 2011);

^d^ KOG Description assigned by the Soybean Genome Project, DoE Joint Genome Institute (http://www.phytozome.net/soybean.php, updated on July 2011);

^e^ CC: Constitutive contrast—significant fold difference in transcript abundance between Conrad and Sloan (C : S) in mock-inoculated samples at specified time points (HAI);

^f^ RC: Response contrast—significant fold differences in infection response ratios (inoculated / mock-inoculated) in Conrad compared to Sloan (C : S) at specified time points (HAI);

^g^ IC: Infection contrast—significant fold difference in transcript abundance between Conrad and Sloan (C : S) in infected samples at specified time points (HAI);

^h^ Samples were collected from the inoculation site at 72 HAI;

^i^ Samples were collected from the front of lesion margin at 72 HAI.

**Table 5:** **Genes with significant expression differences in the R group vs. S group in qRT-PCR.*P***  **< 0.05, Fold difference > 1.5**

**GlymaID**	**PFAM**^**a**^	**GO function**^**b**^	**PANTHER**^**c**^	**KOG**^**d**^	**BLASTP**	**E-value**	**BLAST hit species**	**Contrasts**	**Sampling points (hai)**				
									**12**	**24**	**48**	**72_I**^**h**^	**72_U**^**i**^
Glyma19g35340	Zinc-binding dehydrogenase	Zinc ion binding	Alcohol dehydrogenase related	Alcohol dehydrogenase, class III	Alcohol dehydrogenase, putative	0E + 00	*Ricinus communis*	CC^e^					
								RC^f^				1.5	
								IC^g^					
Glyma19g40800	WD domain, G-beta repeat	-	WD repeat protein	WD-repeat protein WDR6, WD repeat superfamily	Transducin/WD40 domain-containing protein	0E + 00	*Arabidopsis thaliana*	CC					
								RC				1.8	
								IC					
Glyma19g40940	Glycosyl hydrolases family 28	Carbohydrate metabolism; polygalacturonase activity	-	-	Glycoside hydrolase family 28 protein	0E + 00	*Arabidopsis thaliana*	CC					
								RC			−1.9		
								IC			−1.6		
Glyma19g40950	WRKY DNA -binding domain	Transcription factor activity; sequence-specific DNA binding	-	-	Putative WRKY transcription factor 42	9E-91	*Arabidopsis thaliana*	CC					
								RC					
								IC			−1.5		
Glyma19g40970	AUX/IAA family	Transcription factor activity	-	-	Auxin-responsive protein IAA20, putative	1E-48	*Ricinus communis*	CC	−1.5				
								RC					−1.9
								IC		−2.2			−1.8
Glyma19g41580	-	-	-	-	Transcription factor bHLH149	1E-30	*Arabidopsis thaliana*	CC			−1.6	−2.8	
								RC			1.5	2.3	
								IC					
Glyma19g41780	GATA zinc finger	Transcription factor activity; regulation of transcription, DNA-dependent; zinc ion binding; sequence-specific DNA binding	Transcription factor gata (gata binding factor)	-	GATA transcription factor 16	1E-23	*Arabidopsis thaliana*	CC					
								RC			−1.5		
								IC					
Glyma19g41800	Kinesin motor domain	ATPase activity microtubule binding microtubule motor activity	Kinesin heavy chain	Kinesin (KAR3 subfamily)	Kinesin heavy chain, putative	0E + 00	*Ricinus communis*	CC	−5.1	−5.6	−6.7	−2.9	−3.3
								RC				2.0	
								IC	−5.4	−6.2	−7.1	−6.2	−2.7
Glyma19g41870	Protein phosphatase 2C	Protein serine/threonine phosphatase activity	Protein phosphatase 2c	Serine/threonine protein phosphatase	Protein phosphatase 2c, putative	0E + 00	*Ricinus communis*	CC					
								RC			1.6		
								IC					
Glyma19g41900	F-box domain	-	-	-	Phloem-specific lectin PP2-like protein	2E-82	*Arabidopsis thaliana*	CC					
								RC			1.9		
								IC					
Glyma19g41930	Leucine Rich Repeat	Protein binding	F-box/leucine rich repeat protein	Leucine rich repeat proteins, some proteins contain F-box	Ubiquitin-protein ligase, putative	0E + 00	*Ricinus communis*	CC					
								RC				2.1	
								IC					
Glyma19g42050	Calcineurin-like phosphoesterase	Protein serine/threonine phosphatase activity	Serine/threonine protein phosphatase	Serine/threonine specific protein phosphatase PP1, catalytic subunit	Serine/threonine-protein phosphatase PP1 isozyme 8	0E + 00	*Arabidopsis thaliana*	CC					
								RC			1.8		
								IC					
Glyma19g42120	-	-	-	Uncharacterized conserved protein	heparan-alpha-glucosaminide N-acetyltransferase	0 + 00	*Arabidopsis thaliana*	CC					
								RC				2.0	
								IC					
Glyma19g42200	Rapid ALkalinization Factor (RALF)	Signal transducer activity	-	-	Rapid ALkalinization Factor	9e-47	*Medicago truncatula*	CC					
								RC			1.6		
								IC					
Glyma19g42340	Protein tyrosine kinase	Protein-tyrosine kinase activity; protein amino acid phosphorylation; ATP binding	Mapkk-related serine/threonine protein kinases	MEKK and related serine/threonine protein kinases	Mitogen activated protein kinase kinase kinase 3, mapkkk3, mekk3, putative	0E + 00	*Ricinus communis*	CC					
								RC				2.8	
								IC				2.1	2.0

R group: lines with the Conrad haplotype (Conrad, the RILs 2022, and 1960); S group: lines with the Sloan haplotype (Sloan, the RILs 1974, and 2022). Empty cell indicates that there was no significant difference, and a negative value indicates the fold of greater expression ratio in the group with the Sloan haplotype.

^a^ PFAM description provided by the Soybean Genome Project, DoE Joint Genome Institute (http://www.phytozome.net/soybean.php, updated on July 2011);

^b^ Gene Ontology Descriptions obtained from the (http://soybase.org, updated on July 2011);

^c^ PANTHER description provided by the Soybean Genome Project, DoE Joint Genome Institute (http://www.phytozome.net/soybean.php, updated on July 2011);

^d^ KOG Description assigned by the Soybean Genome Project, DoE Joint Genome Institute (http://www.phytozome.net/soybean.php, updated on July 2011);

^e^ CC: Constitutive contrast—significant fold difference in transcript abundance between the group with Conrad haplotype and the group with Sloan haplotype in mock-inoculated samples at specified time points (hai);

^f^ RC: Response contrast—significant fold differences in infection response ratios (inoculated / mock-inoculated) in the group with Conrad haplotype compared to the group with Sloan haplotype at specified time points (hai);

^g^ IC: Infection contrast—significant fold difference in transcript abundance between the group with Conrad haplotype and the group with Sloan haplotype in infected samples at specified time points (hai);

^h^ Samples were collected from the inoculation site at 72 HAI;

^i^ Samples were collected from the front of lesion margin at 72 HAI.

**Figure 4:**
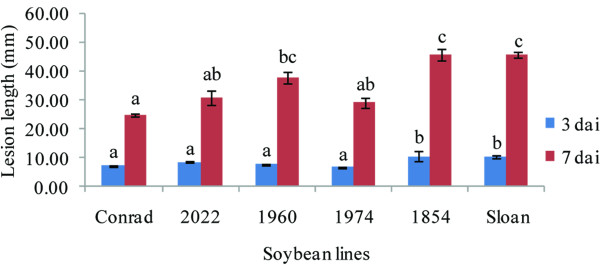
**Lesion lengths of the six lines for qRT-PCR after inoculation with*Phytophthora sojae*.** The data at 3 day after inoculation (dai) were collected from the qRT-PCR assay while the data at 7 dai were collected from the mapping study of the Conrad x Sloan F_6:8_ population. For each time point, bars with different letters indicate the significantly different lesion lengths (*P* < 0.05).

During the qRT-PCR experiments, lesion symptoms were not visible until 72 hai, which was the same timing as symptom development reported in the microarray assays [10,21]. Samples for analysis were collected at the inoculation site for the first three time points. At 72 hai, significantly longer lesions were observed in S and RIL 1854 in comparison to the remaining four lines (*P* < 0.05, Figure 4); tissue samples were collected both above the lesion margin, similar to the microarray assays [10,21,22], and at the inoculation site. Most changes of transcript abundance were observed at 48 and 72 hai, which was similar to the previous findings that most of the transcript abundance changes in the expression of partial resistance to *P. sojae* in soybean occurred 48 hai or later [10,18,22]. Similar expression patterns were obtained with qRT-PCR compared to microarray data for six of the eight genes at all time points. However, Glyma19g42240 and Glyma19g42460 (Histone-like transcription factors) had suppressed transcript levels at 48 hai in microarray assays [10], but not in the qRT-PCR assays.

Overall, sixteen of the genes analyzed with qRT-PCR had significant differences in transcript levels between R and S in either mock-inoculated samples, infected samples, or infection response (*P* < 0.05, fold difference > 1.5, Table 4). These three types of expression contrasts were also analyzed between the three lines harboring the R haplotype (R group, Table 1) and the three lines with the S haplotype (S group, Table 1) with significant differences observed for 15 genes (*P* < 0.05, fold difference > 1.5, Table 5). Significantly different infection responses in 11 genes were observed between R and S, as well as between the R and S group (Tables 4 and 5). Eight of the 11 genes had SNPs in upstream, UTR, exon, intron, or downstream region. The annotated functions and differential expression patterns of these 11 genes suggested their potential association with the higher level of partial resistance in R compared to S.

### Signaling genes

**Figure 5:**
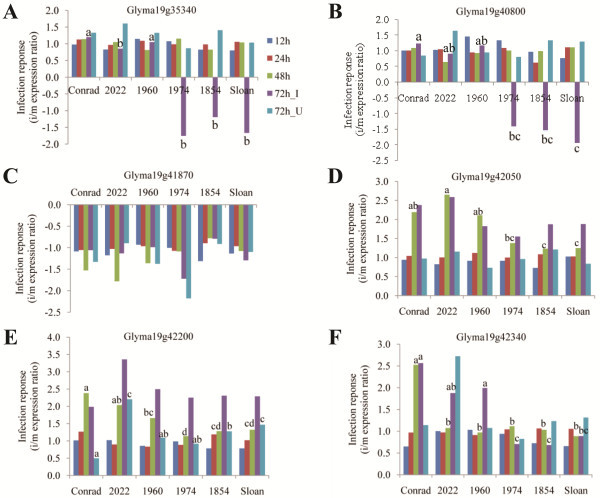
**Signaling genes from QTL 19–1 and 19–2 with significant infection response in qRT-PCR assays.A**. Glyma19g35340 (Alcohol dehydrogenase, class III); **B**. Glyma19g40800 (transducin/WD40 domain-containing protein); **C**. Glyma19g41870 (Protein phosphatase 2C); **D**. Glyma19g42050 (Calcineucin-like phosphatase); **E**. Glyma19g42200 (Rapid alkalinization factor RALF); **F**. Glyma19g42340 (MAP3K-ANP1-like). Bars labeled with different letters indicate the significantly different infection response between samples at a specific time point ( *P* < 0.05). Letters only appear above the bars of time points for which there were significant differences between Conrad and Sloan.

Five genes with annotated functions in signal transduction were found to be associated with the high level of partial resistance in R. Calcineurin-like phosphatase (Glyma19g42050) is a Ca^2+^- and calmodulin-dependent serine/threonine phosphatase. It was up-regulated at 48 hai in the R group, which was 24 h earlier than the S group (Figure 5). This gene is involved in calcium-signaling, which is an important component of plant-pathogen interactions [38]. Repression of calcineurin-like proteins resulted in hypersensitivity to abscisic acid (ABA), indicating their roles as negative regulators of an ABA signaling pathway [39,40]. ABA was also reported to be a negative regulator of *R*-gene mediated resistance against *P. sojae* in soybean through suppression of salicylic acid (SA)-mediated defense pathways [41,42]. The results from this study indicated calcineurin-like phosphatase in the defense pathways could contribute to the high level of partial resistance in R genotype. Its potential interaction with ABA-signaling needs to be further explored in future studies in the soybean partial resistance to *P. sojae*.

MAP3K is part of the MAP kinase cascade, which is known to be one of the early signaling events in PAMP-triggered immunity (PTI). The *MAP3K-ANP1* gene in Arabidopsis was reported to suppress early auxin response but activate MPK3 and MPK6, which are the positive regulators of plant defense in PTI [43,44]. In this study, a *MAP3K-ANP1-like* gene Glyma19g42340 with 11 SNPs in S compared to both R and Williams 82, with seven in the upstream, three in the introns, and one synonymous SNP was identified. The *MAP3K-ANP1-like* gene was up-regulated during infection in the R genotype at 48 and 72 hai. This gene also exhibited significantly higher transcript abundances in the R group than the S group at the inoculation sites 72 hai (Figure 5, Table 5). These transcriptional results make this a candidate gene that may be involved in regulating soybean defense to *P. sojae.*

Class III alcohol dehydrogenase (ADH), is also known as the glutathione-dependent formaldehyde dehydrogenase (FALDH) or S–nitrosoglutathione reductase (GSNOR). It functions in nitric oxide (NO) signaling, which is an important signaling pathway in regulating defense gene expression, defense hormone interplay, and oxidative stress response during plant-pathogen interactions [45-48]. In Arabidopsis the orthologous gene, *ADH2*, has been demonstrated to positively regulate basal resistance to *Pseudomonas syringae* and *Hyaloperonospora parasitica* through activating SA-mediated defense pathway [46]. However, another study reported that down-regulation of ADH2 increased the basal resistance in Arabidopsis to *H. parasitica*[48]*.* In this study, an ADH2 ortholog in soybean (Glyma19g35340) had 19 SNPs in S compared to both R and Williams 82, in the upstream, exon (one of the two SNPs was non-synonymous), intron, 3’ UTR and downstream regions. It was down-regulated at the inoculation sites 72 hai in the S group only (Figure 5). The suppression of this gene may potentially contribute to the susceptibility of soybean to *P. sojae*.

Rapid alkalinization factor (RALF) was first identified in a search for bioactive defense peptides in tobacco, and it was reported to promote extracellular alkalinity and activate MAP kinases [49]. Significantly higher levels of RALF activation were observed in the resistant variety of chickpea in comparison to the susceptible one at 48 hai with *Fusarium oxysporum*[50]. Interestingly, in a study with poplar, methyl jasmonate (MeJA) treatment was found to strongly suppress RALF expression [51], which may indicate that the defense pathways associated with activation of RALF were different than the jasmonic acid (JA)-mediated pathway. In this study, a RALF-encoding gene (Glyma19g42200) had unique sequence in R compared to both S and Willams82 (Table 2). It was up-regulated in the R group at 48 hai, which was 24 h earlier than the S group (Figure 5). These results suggest its potential association with partial resistance in R.

A transducin/WD40 domain-containing protein (Glyma19g40800) had unique sequence in R compared to both S and Williams82 (Table 2). It was down-regulated in the S group only at the inoculation site 72 hai (Figure 5). Members of this class of genes in other plant species have been reported to be up-regulated in the resistant response to pathogen infection, such as in potato against *P. infestans*[52] and in Arabidopsis against *Colletotrichum higginsianum*[53]. The suppression of this gene in soybean may potentially contribute to the susceptibility to *P. sojae*.

### Genes involved in hormone-mediated pathways

**Figure 6:**
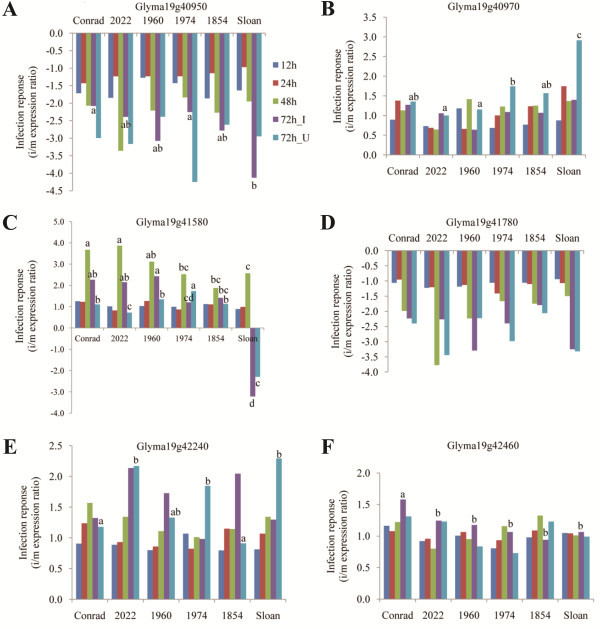
**Transcription factors from QTL 19–2 with significant infection response in qRT-PCR assays.A**. Glyma19g40950 (WRKY transcription factor); **B**. Glyma19g40970 (Auxin-responsive transcription factor); **C**. Glyma19g41580 (putative bHLH transcription factor); **D**. Glyma19g41780 (GATA Zinc-finger transcription factor); **E**. Glyma19g42240 (Histone-like transcription factor); **F**. Glyma19g42460 (Histone-like transcription factor). Bars labeled with different letters indicate the significantly different infection response between samples at a specific time point ( *P* < 0.05). Letters only appear above the bars of time points for which there were significant differences between Conrad and Sloan.

Auxin signaling and transport has been found in several other pathosystems to promote plant susceptibility to bacterial and fungal pathogens [54-56]. In Arabidopsis, PTI suppressed the binding of Auxin-responsive TFs to the promoters of downstream genes, hence down-regulating the auxin-response pathway [57]. In soybean-*P. sojae* interactions, transcript abundance of a PIN1-like auxin transport protein and an auxin-induced protein were both found to be up-regulated in S but were suppressed in R at 3 and 5 dai [10]. In this study, an auxin-responsive TF (Glyma19g40970) was up-regulated only in the S group at the infection front 72 hai (Figure 6). At the same time, the infected samples of S group also had significantly higher transcript abundance than the R group (Table 5). These results suggest the potential role of auxin may be in the susceptible response, and one of the partial resistance mechanisms in R may be the suppression of *P. sojae*-induced auxin-signaling.

A bHLH TF, MYC2, was reported to positively regulate JA response to wounding and insect attack, but negatively regulate the JA response to pathogen infection [58]. This gene has been proposed to be a key regulator in the crosstalk among SA-, JA-, ET-, and ABA-mediated signaling pathways [59,60]. A putative bHLH TF (Glyma19g41580) in this study had one SNP at the 251 bp upstream in R compared to both S and Williams 82, and the other synonymous SNP in an exon. Significantly higher level of up-regulation was observed with this gene in the R group at the inoculation site 48 and 72 hai (Figure 6, Table 5), which again indicated the potential involvement of these hormone-mediated pathways in soybean partial resistance to *P. sojae*.

As observed in previous studies [4], the disease symptoms in this study were first observed in soybean roots at 72 hai with *P. sojae*, which indicated that biotrophic stage of infection occurred at inoculation site during the first 48 hai, while necrotrophic stage can be seen at 72 hai. The genes encoding bHLH and auxin-responsive TFs, together with the calcineurin-like phosphatase encoding gene, MAP3K-ANP1-like gene, and RALF signaling gene discussed earlier, were all induced in R group during biotrophic infection. Based on the functional studies of these genes in different pathosystems combined with the analyses in this study, it indicates the potential involvement of SA-mediated pathway, accompanied by suppression of auxin-, and/or ABA-mediated pathways contributing to the expression of partial resistance to *P. sojae* in soybean. Interestingly, auxin-, ABA-, or JA-signaling have each been reported to work antagonistically with SA against (hemi)biotrophic pathogens, and elevated SA levels will suppress these three hormone-mediated pathways [59,60]. The cross-talk among these hormone-mediated pathways and their contribution to partial resistance in soybean against *P. sojae* should be a focus in future studies.

### Genes involved in modification of plant cell structures

**Figure 7:**
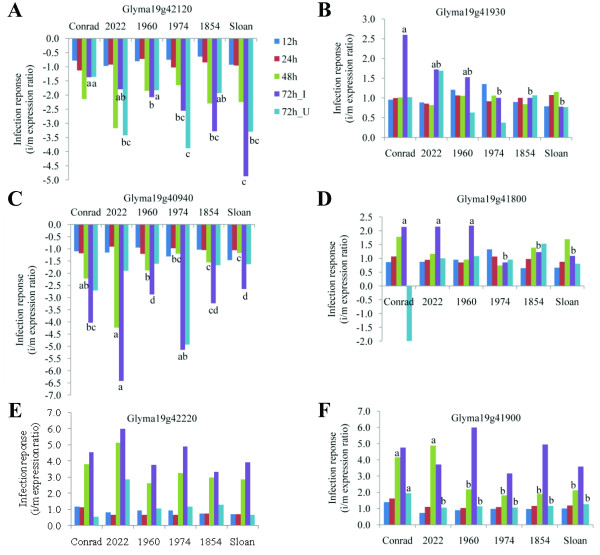
**Genes from different functional categories in QTL 19–2 with significant infection response in qRT-PCR assays.A**. Metabolism: Glyma19g42120 (heparan-alpha-glucosaminide N-acetyltransferase); **B**. Protein modification: Glyma19g41930 (putative ubiquitin-protein ligase); **C**. Cell wall: Glyma19g40940 (Glycosyl hydrolases family 28); **D**. Cytoskeleton: Glyma19g41800 (Kinesin motor); **E**. oxidation: Glyma19g42220 (Calcium-binding oxidoreductase); and **F**. Other: Glyma19g41900 (putative phloem-specific lectin PP2). Bars labeled with different letters indicate the significantly different infection response between samples at a specific time point ( *P* < 0.05). Letters only appear above the bars of time points for which there were significant differences between Conrad and Sloan.

Modification of plant cell structures is an important aspect of plant defense response and three genes, each involved in modification of cell wall, cytoskeleton, and phloem structure, potentially contributed to the expression of partial resistance to *P. sojae*. Glycosyl hydrolase 28 (GH28) hydrolyses pectin is one of the major components of plant cell walls [61-63]. Many bacterial and fungal pathogens secret this enzyme to help them penetrate plant cells [64-66]. In this study, a GH28-encoding gene (Glyma19g40940) had a unique sequence in R as compared to both S and Williams82 (Table 2). Transcription for this gene was down-regulated at 48 hai in the R group, which was 24 h earlier compared to the S group (Figure 7) and may indicate the suppression of cell wall degradation as one of the many components in the expression of partial resistance in soybean against *P. sojae*.

Kinesin motor is one of the cytoskeletal motors, which may participate in defense responses when a plant is challenged by environmental stresses or pathogen attacks [67,68]. A kinesin motor (Glyma19g41800) in this study had a predicted premature stop codon in R, and the resulting peptide is predicted to be missing 650 amino acids from the C-terminus compared to both S and Williams 82 (Table 3, Additional file 4). It had significantly lower transcript abundance in both mock-inoculated and infected samples of the R group than the S group (Tables 4 and 5), possibly due to the predicted truncated protein in R; however, this needs to be validated. In addition, this gene was up-regulated at the inoculation site 72 hai only in the R group (Figure 7), which may indicate the potential involvement of the R allele in soybean partial resistance against *P. sojae*.

Plant lectins belong to a large gene family with diverse functions, one of which is the anti-microbial function in plant defense [69-71]. Soybean cultivars with *R*-gene mediated resistance towards *P. sojae* were found to have two-fold more lectins in seeds than susceptible cultivars [72]. Phloem-specific lectins are also known to be related with defined stages of phloem differentiation [73], and their interaction with mesophyll plasmodesmata are known to increase the size exclusion limit of movement between cells [74]. In this study, a putative phloem-specific lectin PP2 gene (Glyma19g41900) had unique sequence in R compared to both S and Williams82 (Table 2 and 3). It was up-regulated at the inoculation site at 48 and 72 hai across all the resistant and susceptible lines, however, significantly higher fold changes were observed in the R group at 48 hai (Figure 7, Table 5). Thus this gene may also potentially contribute to the higher level of partial resistance of soybean to *P. sojae*.

### Genes involved in ubiquitination

Ubiquitination, which functions in protein modification and degradation, has been found to be an important regulator of plant defense response, such as the oxidative burst, hormone signaling, gene induction, and programmed cell death [75]. Ubiquitin ligases are the key enzymes to select target proteins for ubiquitination. These ligases have been reported to regulate SA-, JA/ET-mediated pathways and they could have either positive or negative effect on plant defense [75,76]. In this study, a putative ubiquitin-protein ligase (Glyma19g41930) was activated at the inoculation site 72 hai in the R group only (Figure 7), which may indicate this gene as a positive regulator of soybean partial resistance against *P. sojae.*

From the 11 genes discussed earlier, three genes, an auxin-responsive TF, a transducin/WD40 domain-containing protein, and a class III alcohol dehydrogenase, all had significant infection response in S group only. Their annotated functions suggested that these responses may potentially contribute to the soybean susceptibility to *P. sojae*. The response of auxin-responsive TF was observed 72 hai at infection front, where biotrophic infection occurred; whereas the other two genes were down-regulated 72 hai at inoculation site, where the necrotrophic phase is in progress. Distinct effector proteins have been found to be secreted by another oomycete pathogen, *P. infestans*, during biotrophic and necrotrophic stages of infection [77]. Thus, secreted effectors from *P. sojae* may also mediate the different stages of infection, potentially targeting these three genes as well as others to suppress the defense response. Further studies are needed to assess if the sequence variation of these genes contributes to the pathogen effector-target recognition process, which in turn contribute to the higher level of partial resistance.

## Conclusions

It is usually difficult to demonstrate the effect of each individual gene underlying a QTL on partial resistance due to the large number of genes with minor and additive effects on the phenotypes [78]. In this study, we utilized sequence and expression analysis to efficiently identify candidate genes underlying the soybean QTL conferring resistance to *P. sojae*. Two QTL were dissected by sequence and gene expression analysis between the resistant and susceptible genotypes. A list of candidate genes was identified, including those potentially involved in signal transduction, hormone-mediated defense pathways, plant cell structural modification, and ubiquitination. Also, several genes from this list have been reported for their roles in PTI in heterologous systems, which may indicate that basal resistance may be another component of partial resistance. These findings supported our hypothesis that defense to *P. sojae* may be a coordinated, multifaceted response to infection. Eleven of the 15 genes with SNPs had significantly different changes in transcript abundance between the R and S genotypes in response to *P. sojae* infection in the qRT-PCR assay, which also supports our hypothesis that SNP analysis could expedite the identification of candidate genes involved in partial resistance. In addition to transcriptional regulation examined in this study, other regulatory mechanisms, including post-transcriptional and translational regulation, could contribute to the differential partial resistance levels between R and S and represent interesting targets for future studies. Whole-genome sequencing of these two cultivars may aid in the discovery of Conrad-specific genes, which may contribute to partial resistance. Overall, this study provides an initial list of candidate genes for further study and additional SNP markers for fine mapping and marker-assisted breeding of soybean partial resistance to *P. sojae*.

## Methods

### Plant resources

An F_6:8_ recombinant inbred line (RIL) population was developed from a cross of soybean cultivar R (Conrad) by S (Sloan). This population was advanced by single seed descent from the F_4:6_ population that was used in the studies of [10,11].

### Inoculum and phenotypic assay

The 246 RILs of the F_6:8_ Conrad × Sloan population were evaluated for the expression of resistance by measuring lesion length following inoculation with *P. sojae* isolate 1.S.1.1 ( *vir* 1a, 1b, 1 k, 2, 3a, 3b, 3c, 4, 5, 6, 7, 8) using the tray test assay, of which the procedure was described in detail previously [6,10,79]. Roots of 7-day-old soybean seedlings were inoculated 20 mm below the crown. Seven dai, the lesion on each seedling was measured from the point of inoculation up to the top of lesion margin. The experimental design was an augmented randomized complete block (RCB), with at least 82 RILs evaluated within each block. The R and S parents were included in each block and there were three blocks within each experiment. Each RIL was evaluated three times in three separate experiments. For phenotypic data analysis, BLUP values of each RIL was obtained using a mixed model analysis with the mean lesion length of the 10 plants in each tray, as described in [6,9-11]. Heritability, on a family mean basis, was calculated as described in [10].

### QTL mapping

DNA from each RIL was extracted using the same method as [10]. For this population, 147 RILs were randomly selected and genotyped using the Illumina BeadXpress® Assay (Illumina Inc., San Diego, CA) according to manufacturer’s protocol. DNA samples were first quantified with Picogreen® dsDNA quantification kit (Invitrogen Inc., Carlsbad, CA) and ~250 ng each (50 ng/ul) was used for BeadXpress genotyping, including several activation and ligation steps followed by PCR, hybridization to SNP-specific beads, washing, and plate scanning at the Molecular Cellular Imaging Center (MCIC, OARDC, Wooster, OH). The genotype data was analyzed using the Genome Studio Software® (Illumina Inc., San Diego, CA). A total of 151 SNP markers [33,35,80] were used to build the genetic map using JoinMap ® 4.0 with the Kosambi function [81]. A preliminary analysis with interval mapping to identify potential QTL on 147 RILs in response to *P. sojae* inoculation with MAPQTL® 5.0 [82] and single marker association with one-way ANOVA (Proc GLM, SAS 9.1.3, SAS Institute Inc. Cary, NC) was done.

A total of 57 SSR and 32 SNP markers which targeted the potential QTL regions were genotyped on the 246 RILs. For SNP genotyping, a modified PCR Amplification of Multiple Specific Alleles (PAMSA) technique was used [37]. The procedures of SNP and SSR genotyping were as described in [11]. The genetic map for these potential QTL regions was re-constructed using JoinMap® 4.0 with the Kosambi function [81]. Interval mapping (IM) and composite interval mapping (CIM) of QTL were performed using MAPQTL® 5.0 [82]. The walking speed for QTL analyses was 1.0 centimorgan (cM). Permutation tests with 1000 iterations were performed on each linkage group and on the whole genome to estimate significant LOD scores [83].

### Functional categorization of genes underlying the QTL

Genes underlying the QTL were categorized into 14 groups based on their functional annotations from NCBI BLASTP search and four other databases (GO Function, PFAM, PANTHER, and KOG) [23]. Grouping criteria, modified from [84], included: 1) Signal transduction, which involves calcium signaling genes, G proteins, kinases and phosphatases, and other signal transducers; 2) Metabolism, including genes in both primary and secondary metabolic pathways; 3) Unknown, including genes with no annotations or no characterized functions from all mentioned databases; 4) Protein modification, including genes involved in proteins synthesis, degradation and other structural modification processes; 5) Transcription factor; 6) Transporter; 7) Cell wall, which includes genes in synthesis and modification of different cell wall components; 8) RNA regulation, which involves RNA-binding genes; 9) Energy, which includes genes associated with ATP and electron transfer; 10) Stress response; 11) Cytoskeleton, which involves actin, kinesin, and microtubule-related genes; 12) Oxidation, which includes genes encoding enzymes involved in oxidation; and 13) Pathogenesis-related (PR) protein; and 14) Others, which includes genes not in the previously mentioned categories.

### Long-range PCR (LR-PCR)

Gene sequences were extracted from the soybean reference genome which was generated from the cultivar Williams82 [23]. A total of 217 pairs of gene-specific primers were designed using BatchPrimer3 [85] for 186 genes underlying the QTL 19–1 and 19–2. For each gene, a 1.2 kb upstream region and a 400 bp downstream region were included for primer design. LR-PCR was performed with a 30 μl PCR reaction which contained 30 ng of genomic DNA template, 1 x Phusion HF buffer (New England Biolabs Inc., Ipswich, MA.), 200 μM dNTPs, 0.4 μM forward and reverse primers, and 0.6 U of Phusion® High-Fidelity DNA Polymerase (New England Biolabs Inc., Ipswich, MA.). PCR reactions were performed using the following conditions: 98°C for 2 min, 35 cycles of 98°C for 10 sec, (lower Tm calculated by the nearest neighbor method + 3)°C for 30 sec, and 72°C for 6 min, followed by a final extension at 72°C for 10 min. PCR products were purified by the E-Gel® Clonewell 0.8% SYBR Safe^TM^ agarose and 2% SizeSelect^TM^ agarose (Invitrogen Inc., Carlsbad, CA), and the Zymoclean^TM^ Gel DNA Recovery kit (ZymoResearch Inc., Irvine, CA). The purified PCR products were quantified in 2% agarose gel with ethidium bromide staining. Equal amounts (30 ng) of each PCR product amplified from R and S cultivars were pooled separately and precipitated with 100% EtOH to remove the fluorescent dyes which bound to DNA from the E-gels. The purified PCR product pools were quantified again in 2% agarose gel prior to library construction.

### Library construction for Illumina GA II sequencing

Approximately 3 μg of combined PCR products from R or S were used for library preparation. PCR products were digested with NEBNext dsDNA Fragmentase (New England Biolabs Inc., Ipswich, MA.) according to manufacturer’s instructions. Reactions were carried out in a total volume of 60 μl with 6 μl of fragmentase and incubated in a 37  C water-bath for 25 minutes. The reactions were cleaned using QIAQuick PCR Purification Kit (Qiagen, Valencia, CA). Fragmented DNA was used for Illumina Paired-End (PE) library preparation, using the PE library preparation kit (Illumina Inc., San Diego, CA) as instructed in the manual. To reduce the over-representation of the amplicon ends in sequencing [86], a 400-bp library instead of a standard 200-bp one was constructed. The fragments were end-repaired and phosphorylated using T4 DNA polymerase, Klenow DNA polymerase and T4 PNK and were 3' adenylated using Klenow Exo- (3' to 5' exo minus). Illumina PE adapters were ligated using DNA Ligase, followed by purification on a 2% TAE-agarose gel (Certified Low-Range Ultra Agarose, Biorad). A band of 400 ± 25 bp was cut and purified, using QIAQuick Gel Extraction Kit (Qiagen, Valencia, CA). Enrichment of adapter ligated fragment and the addition of sequences necessary for flow cell binding was done by performing fifteen rounds of PCR, using Illumina PE 1.0 nd PE 2.0 primers. DNA fragment size distribution in the libraries was done with an Agilent Technologies 2100 Bioanalyzer using the Agilent DNA 1000 chip kit. The libraries were quantified, using quantitative PCR with PhiX sequencing control as a standard (Illumina, San Diego, CA). PE sequencing was done, using the Illumina GAII platform at MCIC (OARDC, Wooster, OH).

### Sequence data analyses

Initial quality assessment of sequence reads was performed using Fastqc. Sequence reads with poor quality were filtered (adaptive_qualitytrim.pl). The pre-processed FASTQ files were aligned using the MOSAIKALIGNER set of tools (version 0.9.0891 of the MOSAIK Software Suite; (http://bioinformatics.bc.edu/marthlab/Mosaik). All reads were aligned to the Chr. 19 sequences from the soybean reference genome [23]. SNPs between the reference sequence (Williams82) and samples were identified using Partek Genomics Suite version 6.5 (Partek, St Louis, MO). False-positive SNPs which located outside the amplicons and/or had less than 20 X coverage were removed. The alignment data from the R and S are available at NCBI Sequence Read Archive (SRA) under accession SRA056409.

### SNP verification

Twenty-nine SNPs were selected for verification by PAMSA technique [3], based on the 0.1 Mb interval and predicted gene function. The SNP genotyping procedure was as described in [11]. For one SNP that was not verified by PAMSA, the PCR products from SNP genotyping were purified by Zymoclean^TM^ Gel DNA Recovery kit (ZymoResearch Inc., Irvine, CA) and sent for Sanger sequencing at MCIC (OARDC, Wooster, OH).

### qRT-PCR assays

The tray test protocol was also used for the qRT-PCR time course assay. *P. sojae* isolate 1.S.1.1 was used to inoculate R, S, and four selected RILs with different combination of R or S haplotypes at QTL (Table 1). Samples were collected at 12, 24, 48, and 72 hai. At the first three time points, the inoculation site was sampled; and at 72 hai, lesions were measured and the 0.75 mm from the edge of lesion margin and above was sampled for RNA extraction. For control, mock-inoculated tissues were sampled at the same site as the inoculated samples at each time point. Samples were frozen in liquid nitrogen immediately after collection. The whole assay was repeated once, with two trays per replicate, 10 seedlings per tray. Plant tissue samples collected from all the trays per treatment were pooled for each biological replicate. RNA preparation, cDNA synthesis, and qPCR procedures were as described in [10], except the SuperScript® III First-Strand Synthesis System was used instead (Invitrogen Inc., Carlsbad, CA). The same house-keeping genes used in [10] were used in this study: a putative ubiquitin gene (Gma.441.1.S1_at) and a putative F-box protein (Gma.6079.1.S1_at). Nineteen candidate genes from the QTL 19–1 and 19–2 regions were selected based on their annotated functions, sequence variation between R and S, and available microarray data [10,22]. PCR efficiency (E) of each primer pairs can be estimated from standard curves with the equation (1 + E) =10^(−1/slope)^[87]. Levels of transcript abundance were calculated using the equation (1 + E)^(−ΔCt)^, where ΔCt equaled the value when the average Ct value of the reference genes was subtracted from the Ct value of target gene. Infection response of a target gene was represented by the transcript level fold differences in inoculated (i) samples relative to mock (m) controls, which was calculated from the equation: (1 + E_gene_)^(C_Tm_ - C_Ti_) / Avg((1 + E_ref_)^(C_Tm_ - C_Ti_)), where ref indicated a house keeping gene and Avg was the average of two house-keeping genes. Contrasts of LSMeans were performed among the six soybean lines, or between the three lines with the R haplotype (R group) and those with the S haplotype (S group) for three types of comparisons (SAS 9.2, SAS Institute Inc. Cary, NC): 1) infection response; 2) transcript abundance at mock-inoculated samples; and 3) transcript abundance at inoculated samples. The significant differences of these comparisons were determined by: 1) *P* < 0.05; 2) fold difference > 1.5.

## Competing interests

The authors declare that they have no competing interests.

## Authors’ contributions

HW, AW, and AED conceived and designed the experiments; HW and SL performed the experiments; LMcH contributed to marker selection and mapping analysis; SKSt.M developed populations; HW, AW, SW contributed to analysis; HW, CT, LMcH, and AD wrote the article**.** All authors read and approved the final manuscript.

## Supplementary Material

Additional file 1 Genes underlying QTL 19–1 with predicted functions and microarray data. Click here for file

Additional file 2 Genes underlying QTL 19–2 with predicted functions and microarray data. Click here for file

Additional file 3 SNPs detected between Conrad and Sloan in sequenced genes underlying QTL 19–1 and 19–2. Click here for file

Additional file 4 Sequence polymorphisms in Conrad (C) in comparison to Williams82 (W) and Sloan (S) in Glyma19g41420 and Glyma19g41800, respectively. Click here for file
