# Supplementary material for: Dissection of two soybean QTL conferring partial resistance to Phytophthora sojae through sequence and gene expression analysis
Source: BMC Genomics. 2012 Aug 28;13:428. doi: 10.1186/1471-2164-13-428 (PMC3443417; doi:10.1186/1471-2164-13-428)
Supplement: Additional file 1 — Genes underlying QTL 19–1 with predicted functions and microarray data. [file 1471-2164-13-428-S1.doc]

Additional file 1. Genes underlying QTL 19-1 with predicted functions and microarray data.

| **Gene** | **PFAM^a^** | **GO function^b^** | **PANTHER^c^** | **KOG^d^** | **Affy ID^e^** | **IR^f^_C** | **IR_S** |
| --- | --- | --- | --- | --- | --- | --- | --- |
| Glyma19g35270.1 | ABC transporter | ATPase activity, coupled to transmembrane movement of substances | ATP-binding cassette transporter | Pleiotropic drug resistance proteins (PDR1-15), ABC superfamily | Gma.16908.1.S1_at | - | -3 |
|  |  |  |  |  | GmaAffx.5080.1.A1_at | - | -2 |
|  |  |  |  |  | GmaAffx.5080.1.S1_at | - | - |
| Glyma19g35280.1 | WD domain, G-beta repeat | Nucleotide binding | Gastrulation defective protein 1-related | Uncharacterized conserved protein, contains WD40 repeat | GmaAffx.63550.1.S1_at | - | - |
| Glyma19g35290.5 | Mediator complex protein | RNA polymerase II transcription mediator activity/Srb-mediator complex/regulation of transcription from Pol II promoter | - | - | Gma.10825.1.S1_at | -2,-5 | -3,-5 |
| Glyma19g35300.1 | - | - | VHS domain containing protein family | - | GmaAffx.650.2.S1_at | No data | No data |
| Glyma19g35320 | Unknown | - | - | - | - |  |  |
| Glyma19g35330 | - | - | Suppression of tumorigenicity 5 (st5) | - | - |  |  |
| Glyma19g35340 | Zinc-binding dehydrogenase | Zinc ion binding | Alcohol dehydrogenase related | Alcohol dehydrogenase, class III | - |  |  |
| Glyma19g35350 | Plus-3 domain | Transcription initiation | - | Paf1/RNA polymerase II complex, RTF1 component (involved in regulation of TATA box-binding protein) | - |  |  |
| Glyma19g35360 | Protein of unknown function (DUF861) | - | - | - | - |  |  |
| Glyma19g35370.2 | WD domain, G-beta repeat | - | Family not named | WD40 repeat-containing protein | GmaAffx.68845.1.S1_at | - | 2 |
| Glyma19g35380.2 | WD domain, G-beta repeat | Nucleotide binding | WD repeat protein 26-related | WD40 repeat-containing protein | GmaAffx.87976.1.A1_at | - | - |
| Glyma19g35390.1 | Protein tyrosine kinase | Kinase activity | Serine-threonine protein kinase, plant-type | Serine/threonine protein kinase | GmaAffx.31697.1.S1_at | -2 | -2,5 |
| Glyma19g35410 | DIL domain/Myosin head (motor domain/IQ calmodulin-binding motif/Myosin N-terminal SH3-like domain | - | Myosin | Myosin class V heavy chain | - |  |  |
| Glyma19g35420 |  |  | Dimethylaniline monooxygenase | Flavin-containing monooxygenase | - |  |  |
| Glyma19g35430 | Protein of unknown function (DUF3741) | - | Phosphatidylinositol n-acetylglucosaminyltransferase subunit p (down syndrome critical region protein 5)-related | - | - |  |  |
| Glyma19g35450.1 | Leucine rich repeat | Protein binding | nalp (nacht, leucine rich repeat and pyrin domain containing)-related | Ran gtpase-activating protein | Gma.7151.1.S1_s_at | - | -3 |
|  |  |  |  |  | GmaAffx.24365.1.A1_at | - | -3 |
| Glyma19g35460 | HEAT repeat | - | CLASP | CLIP-associating protein | - |  |  |
| Glyma19g35470 | Response regulator receiver domain | Two-component response regulator activity | Sensor histidine kinase-related | - | - |  |  |
| Glyma19g35480 | Response regulator receiver domai | Two-component response regulator activity | Sensor histidine kinase-related | - | - |  |  |
| Glyma19g35490 | - | - | Ankyrin repeat-containing | - | - |  |  |
| Glyma19g35510.1 | Atpase family associated with various cellular activities (AAA) | ATP binding | 26s protease regulatory subunit | 26S proteasome regulatory complex, atpase RPT2 | Gma.2905.1.S1_at | - | - |
| Glyma19g35520.1 | - | - | - | - | Gma.5496.1.S1_at | No data | No data |
| Glyma19g35530.1 | - | - | - | - | Gma.15735.1.A1_at | No data | No data |
| Glyma19g35540 | - | - | - | - | - |  |  |
| Glyma19g35550.2 | - | - | - | - | GmaAffx.15146.1.S1_at | 2 | 2 |
| Glyma19g35560 | Hsp70 protein | - | Heat shock protein 70kda | Molecular chaperones GRP78/bip/KAR2, HSP70 superfamily | - |  |  |
| Glyma19g35570.1 | - | - | - | - | Gma.11179.3.S1_at | - | -2 |
| Glyma19g35580 | - | - | - | - | - |  |  |
| Glyma19g35590 | Oligosaccaryltransferase | - | - | - | - |  |  |
| Glyma19g35610 | - | - | - | - | - |  |  |
| Glyma19g35620.1 | Putative gtpase activating protein for ARF | Regulation of ARF GTPase activity | Centaurin/ARF | ADP-ribosylation factor gtpase activator | Gma.8559.1.S1_at | -3,-5 | -3,-5 |
| Glyma19g35630.1 | Short chain dehydrogenase | Oxidoreductase activity | Short-chain dehydrogenases/reductase | Dehydrogenases with different specificities (related to short-chain alcohol dehydrogenases | GmaAffx.25220.1.S1_at | - | - |
| Glyma19g35640.1 | - | - | - | - | GmaAffx.10569.1.A1_at | No data | No data |
| Glyma19g35650.1 | Major facilitator superfamily | Transmembrane transport | UNC-93 related | Predicted membrane protein | GmaAffx.45885.1.S1_at | -2,-3,-5 | -2 |
| Glyma19g35660.1 | PCI domain | - | Family not named | - | GmaAffx.3983.1.A1_s_at | - | - |
| Glyma19g35670 | RNA recognition motif. (a.k.a. RRM, RBD, or RNP domain | Nucleic acid binding | RNA-binding protein | - | - |  |  |
| Glyma19g35680.1 | - | - | - | - | Gma.10906.1.S1_at | -2,-3,-5 | -2,-3,-5 |
| Glyma19g35690.1 | Core histone H2A/H2B/H3/H4 | DNA binding | Histone H2B | Histone H2B | Gma.5572.1.S1_s_at | -2 | -2,3 |
|  |  |  |  |  | GmaAffx.6522.1.A1_at | 5 | 3,5 |
| Glyma19g35700.1 | - | - | - | - | Gma.8157.1.S1_at | 3 | 3,5 |
| Glyma19g35710 | Inorganic pyrophosphatase | Magnesium ion binding | Inorganic pyrophosphatase | Inorganic pyrophosphatase/Nucleosome remodeling factor, subunit NURF38 | - |  |  |
| Glyma19g35720 | Integral membrane protein DUF6 | Membrane | - | - | - |  |  |
| Glyma19g35730.1 | Endonuclease/Exonuclease/phosphatase family | - | Inositol 5-phosphatase | Inositol polyphosphate 5-phosphatase and related proteins | GmaAffx.81708.1.S1_at | -2,-3,-5 | -2,-3,-5 |
| Glyma19g35740.1 | Zinc finger, C2H2 type, dsrna-binding | Intracellular | Zinc finger protein | - | GmaAffx.7139.1.S1_at | 2,3,5 | 2,3,5 |
| Glyma19g35770.1 | Zinc finger, C2H2 type, dsrna-binding | Intracellular | Zinc finger protein | - | GmaAffx.1301.28.S1_at | 2 | 2 |
| Glyma19g35780 | Diaminopimelate epimerase | Cytoplasm | - | - | - |  |  |
| Glyma19g35790 | Protein of unknown function (DUF1635) | - | - | - | - |  |  |
| Glyma19g35800 | Protein kinase domain | Protein kinase activity | CDC2-related kinase | LAMMER dual specificity kinases | - |  |  |
| Glyma19g35810 | Plant protein 1589 of unknown function | - | - | - | - |  |  |
| Glyma19g35820 | Tetratricopeptide repeat | - | O-linked n-acetylglucosamine transferase, ogt | - | - |  |  |
| Glyma19g35840 | - | - | N-acetylglucosaminyltransferase subunit p (down syndrome critical region protein 5)-related | - | - |  |  |
| Glyma19g35870.1 | Heavy-metal-associated domain | Metal ion transport | Copper transport protein atox1-related | Copper chaperone | Gma.18029.1.S1_at | - | - |
| Glyma19g35880 | Exo70 exocyst complex subunit | Exocyst | Exocyst complex protein exo70-related | Exocyst component protein and related proteins | - |  |  |
| Glyma19g35890 | Ankyrin repeat | - | Ankyrin repeat-containing | 26S proteasome regulatory complex, subunit PSMD10 | - |  |  |

| ^a^: PFAM description provided by the Soybean Genome Project, DoE Joint Genome Institute ( <http://www.phytozome.net/soybean.php>, updated on July 2011); |
| --- |
| ^b^: Gene Ontology Descriptions obtained from the ([http://soybase.org](http://soybase.org/), updated on July 2011); |
| ^c^: PANTHER description provided by the Soybean Genome Project, DoE Joint Genome Institute (<http://www.phytozome.net/soybean.php>, updated on July 2011); |
| ^d^: KOG Description assigned by the Soybean Genome Project, DoE Joint Genome Institute (http://www.phytozome.net/soybean.php, updated on July 2011);  ^e^: Affymetrix probe IDs that match with the predicted genes underlying QTL (<http://soybase.org/AffyChip/>), where - means there was no Affymetrix IDs available for the specific gene;  ^f^: IR: Infection response—significant differences in transcript abundance of inoculated samples compared to mock-inoculated samples in Conrad (C) or Sloan (S) (microarray data, TST-FDR, *P* < 0.05) at specified time points (dai); where - means there was no significant response observed at any time point after inoculation, a positive value indicates that the gene was up-regulated at the specified time point, and a negative value indicates the gene was down-regulated at the specified time point. |
