# Supplementary material for: Dissection of two soybean QTL conferring partial resistance to Phytophthora sojae through sequence and gene expression analysis
Source: BMC Genomics. 2012 Aug 28;13:428. doi: 10.1186/1471-2164-13-428 (PMC3443417; doi:10.1186/1471-2164-13-428)
Supplement: Additional file 2 — Genes underlying QTL 19–2 with predicted functions and microarray data. [file 1471-2164-13-428-S2.doc]

Additional file 2. Genes underlying QTL 19-2 with predicted functions and microarray data.

| **Gene** | **PFAM^a^** | **GO function^b^** | **PANTHER^c^** | **KOG^d^** | **Affy ID^e^** | **IR^f^_C** | **IR_S** |
| --- | --- | --- | --- | --- | --- | --- | --- |
| Glyma19g40800 | WD domain, G-beta repeat | - | WD repeat protein | WD-repeat protein WDR6, WD repeat superfamily | - |  |  |
| Glyma19g40810.1 | S-adenosylmethionine synthetase, N-terminal domain | Copper ion binding methionine adenosyltransferase activity | S-adenosylmethionine synthetase | S-adenosylmethionine synthetase | Gma.406.4.S1_s_at | -2 | -2 |
| Glyma19g40820 | Protein kinase domain | ATP binding; protein kinase activity; protein amino acid phosphorylation | Serine-threonine protein kinase, plant-type | Serine/threonine protein kinase | - |  |  |
| Glyma19g40830.2 | Triose-phosphate Transporter family | Organic anion transmembrane transporter activity | Solute carrier family 35 | Glucose-6-phosphate/phosphate and phosphoenolpyruvate/phosphate antiporter | GmaAffx.37957.1.A1_at | -2 | -2,-3 |
| Glyma19g40840 | Pectinesterase/Plant invertase/pectin methylesterase inhibitor | Pectinesterase activity; cell wall; cell wall modification | - | - | - |  |  |
| Glyma19g40850.1 | PCRF domain|Peptidyl-tRNA hydrolase domain | Translation release factor activity | Peptide chain release factor | Mitochondrial polypeptide chain release factor | Gma.3427.1.S1_at | - | - |
| Glyma19g40860 | Unknown | - | - | - | - |  |  |
| Glyma19g40870 | PPR repeat | - | Pentatricopeptide repeat-containing protein | - | - |  |  |
| Glyma19g40880 | Unknown | - | - | - | - |  |  |
| Glyma19g40890.1 | Ion channel|Ion channel | Outward rectifier potassium channel activity | Potassium channel, subfamily k | - | Gma.12613.1.A1_at | - | - |
| Glyma19g40900.1 | Transferase family | Transferase activity | - | - | GmaAffx.79229.1.S1_at | -2,-3 | -2,-3 |
| Glyma19g40910.1 | N-terminal domain of NEFA-interacting nuclear protein NIP30 | - | Nefa-interacting nuclear protein nip30 | Uncharacterized conserved protein | Gma.9172.1.S1_at | - | - |
| Glyma19g40920 | Sec23/Sec24 helical domain | COPII vesicle coat; protein binding; intracellular protein transport | Protein transport protein sec23 | Vesicle coat complex COPII, subunit SEC23 | - |  |  |
| Glyma19g40930.4 | Prolyl-tRNA synthetase, C-terminal/tRNA synthetase class II core domain (G, H, P, S and T)/Anticodon binding domain | ATP binding aminoacyl-tRNA ligase activity | tRNA synthetase-related | Prolyl-tRNA synthetase | GmaAffx.7362.1.S1_at | -1,-2 | -2,5 |
| Glyma19g40940 | Glycosyl hydrolases family 28 | Carbohydrate metabolism; polygalacturonase activity | - | - | - |  |  |
| Glyma19g40950.2 | WRKY DNA -binding domain | Transcription factor activity; sequence-specific DNA binding | - | - | GmaAffx.21523.2.S1_at | -2,-3,-5 | -2,-3,-5 |
| Glyma19g40960.1 | Chitinase class I | Chitinase activity | Chitinase-related | Predicted chitinase | GmaAffx.29692.1.S1_at | 2,3,5 | 2,3,5 |
|  |  |  |  |  | GmaAffx.29692.2.S1_at | 2,3 | 2,3,5 |
| Glyma19g40970.2 | AUX/IAA family | Transcription factor activity | - | - | Gma.10239.2.A1_at | No data | No data |
| Glyma19g40980.2 | Helix-loop-helix DNA-binding domain | DNA binding protein binding transcription factor activity transcription regulator activity | Circadian protein clock/ARNT/BMAL/PAS | Transcriptional repressors of the hairy/E(spl) family (contains HLH) | Gma.1773.1.A1_at | - | - |
| Glyma19g40990 | Snf7 | Protein transport | Snf7 - related | Uncharacterized conserved protein predicted to be involved in protein sorting | - |  |  |
| Glyma19g41010.1 | MYB-like DNA-binding domain | DNA binding transcription factor activity | MYB-related | Transcription factor, MYB superfamily | GmaAffx.6306.4.S1_at | - | -3 |
| Glyma19g41020 | Unknown | - | - | - | - |  |  |
| Glyma19g41030.1 | Pectinacetylesterase | Carboxylesterase activity | Notum-related | Pectin acetylesterase and similar proteins | Gma.15532.1.S1_at | -2 | -2 |
| Glyma19g41040.1 | Haloacid dehalogenase-like hydrolase | Hydrolase activity | 2-deoxyglucose-6-phosphate phosphatase 2 | Haloacid dehalogenase-like hydrolase | Gma.13710.1.A1_at | No data | No data |
| Glyma19g41050.1 | Cyclophilin type peptidyl-prolyl cis-trans isomerase/CLD | Peptidyl-prolyl cis-trans isomerase activity | Cyclophilin | Peptidyl-prolyl cis-trans isomerase | Gma.10843.2.A1_at | -2,5 | -2,5 |
| Glyma19g41060 | Protein phosphatase 2C | - | Protein phosphatase 2c | - | - |  |  |
| Glyma19g41070 | Cupin | Nutrient reservoir activity | - | - | - |  |  |
| Glyma19g41080 | Pollen allergen; Rare lipoprotein A (RlpA)-like double-psi beta-barrel | - | - | - | - |  |  |
| Glyma19g41090.1 | - | hydrolase activity, hydrolyzing O-glycosyl compounds | - | - | GmaAffx.58409.1.A1_at | No data | No data |
| Glyma19g41100 | Bacterial transferase hexapeptide (three repeats) | - | Sugar-1-phosphate guanyl transferase | GDP-mannose pyrophosphorylase/mannose-1-phosphate guanylyltransferase | - |  |  |
| Glyma19g41110.1 | Unknown | - | - | - | Gma.16854.1.A1_at | - | 5 |
| Glyma19g41120.1 | Peptidase family C1 propeptide|Papain family cysteine protease | - | Cysteine protease family c1-related | Cysteine proteinase Cathepsin L | Gma.5028.1.S1_s_at | - | - |
|  |  |  |  |  | Gma.5028.1.S1_x_at | - | - |
| Glyma19g41130 | WD domain, G-beta repeat | - | F-box and wd40 domain protein | G-protein beta subunit-like protein (contains WD40 repeats) | - |  |  |
| Glyma19g41140.1 | Unknown | - | - | - | Gma.5573.1.S1_at | -2,-5 | -2,-3,-5 |
| Glyma19g41150.1 | DEAD/DEAH box helicase|Helicase conserved C-terminal domain|GUCT (NUC152) domain|Zinc knuckle | ATP binding ATP-dependent helicase activity | Dead box atp-dependent rna helicase | ATP-dependent RNA helicase | Gma.3106.1.S1_at | -2 | -3 |
|  |  |  |  |  | GmaAffx.69356.1.S1_at | - | - |
| Glyma19g41160.1 | Domain of unknown function | - | Organic solute transporter-related | Predicted seven transmembrane receptor - rhodopsin family | GmaAffx.80060.1.A1_at | -3 | -3 |
| Glyma19g41170 | Unknown | - | - | - | - |  |  |
| Glyma19g41180.1 | Unknown | - | - | - | Gma.737.1.A1_at | No data | No data |
| Glyma19g41190 | Elongation factor Tu domain 2 | GTP binding | Translation factor | Mitochondrial translation initiation factor 2 (IF-2; GTPase) | - |  |  |
| Glyma19g41200.1 | - | Molecular function | - | - | GmaAffx.39254.1.A1_at | -5 | - |
| Glyma19g41210.1 | PAS fold|GAF domain|Phytochrome region|Histidine kinase-, DNA gyrase B-, and HSP90-like ATPase | G-protein coupled photoreceptor activity protein histidine kinase activity signal transducer activity red or far-red light photoreceptor activity | Sensor histidine kinase-related | - | GmaAffx.88161.1.S1_at | - | -2,-3,-5 |
| Glyma19g41220 | Cupin domain | - | - | - | - |  |  |
| Glyma19g41230 | POT family | Oligopeptide transport; membrane; transporter activity | Oligopeptide transporter-related | H+/oligopeptide symporter | - |  |  |
| Glyma19g41240 | Pectinacetylesterase | - | Notum-related | Pectin acetylesterase and similar proteins | - |  |  |
| Glyma19g41250.1 | MYB-like DNA-binding domain|Myb-like DNA-binding domain | DNA binding transcription factor activity | MYB-related | Transcription factor, MYB superfamily | GmaAffx.28927.3.S1_at | -3 | -3 |
| Glyma19g41260 | Unknown | - | - | - | - |  |  |
| Glyma19g41270.1 | Unknown | - | - | - | Gma.10465.1.S1_at | -3,-5 | -3,-5 |
| Glyma19g41300 | Unknown | - | - | - | - |  |  |
| Glyma19g41310.1 | G-patch domain|Zinc finger, C2H2 type | Nucleic acid binding | Zinc finger-containing | - | GmaAffx.3741.1.S1_at | - | - |
|  |  |  |  |  | GmaAffx.3741.2.S1_at | -5 | -3,-5 |
| Glyma19g41320.2 | Eukaryotic aspartyl protease; Saposin-like type B | Lipid metabolism | Aspartyl proteases | Aspartyl protease | Gma.2063.1.S1_at | -3,-5 | -2,-3,-5 |
| Glyma19g41330 | - | - | Cysteine desulfurylase | Cysteine desulfurase NFS1 | - |  |  |
| Glyma19g41350 | Pectinesterase; Plant invertase/pectin methylesterase inhibitor | Pectinesterase activity; cell wall; cell wall modification | - | - | - |  |  |
| Glyma19g41360.1 | Protein of unknown function, DUF88 | Molecular function | - | - | Gma.6930.1.S1_at | -3,-5 | -3,-5 |
| Glyma19g41370 | X8 domain | - | - | - | - |  |  |
| Glyma19g41380 | Domain of unknown function (DUF3635) | - | - | Serine/threonine kinase (haspin family) | - |  |  |
| Glyma19g41390 | - | - | ATP-citrate synthase | - | - |  |  |
| Glyma19g41400.2 | Sec23/Sec24 beta-sandwich domain; Sec23/Sec24 helical domain; Sec23/Sec24 zinc finger; Gelsolin repeat; Sec23/Sec24 trunk domain | Transporter activity | SEC24-related | - | Gma.1659.2.S1_a_at | - | - |
| Glyma19g41410 | Unknown | - | - | - | - |  |  |
| Glyma19g41420.2 | Protein kinase domain | Protein kinase activity protein serine/threonine kinase activity | CDC2-related kinase | Glycogen synthase kinase-3 | Gma.10350.1.A1_at | -2 | -2 |
| Glyma19g41420.3 | Protein kinase domain | Protein kinase activity protein serine/threonine kinase activity | CDC2-related kinase | Glycogen synthase kinase-3 | GmaAffx.6924.1.S1_at | -2 | -2,5 |
| Glyma19g41430 | Glycosyl hydrolases family 28 | Carbohydrate metabolism; polygalacturonase activity | - | - | - |  |  |
| Glyma19g41440.3 | - | - | Protease family c15 pyroglutamyl-peptidase i-related | Predicted pyroglutamyl peptidase | Gma.6836.1.A1_at | -2 | - |
|  |  |  |  |  | Gma.6836.1.A1_s_at | -2 | -2,-5 |
| Glyma19g41450.1 | Glucose-6-phosphate dehydrogenase, NAD binding domain| | Glucose-6-phosphate dehydrogenase activity | Glucose-6-phosphate 1-dehydrogenase (g6pd) | Glucose-6-phosphate 1-dehydrogenase | GmaAffx.67847.1.S1_at | -2,-3 | -2,-3,-5 |
|  |  |  |  |  | GmaAffx.67847.2.S1_at | -2,-5 | -2,-5 |
| Glyma19g41460 | Unknown | - | - | - | - |  |  |
| Glyma19g41470.1 | GDSL-like Lipase/Acylhydrolase | Carboxylesterase activity | Zinc finger fyve domain containing protein | - | GmaAffx.17013.1.S1_at | -2 | -2 |
| Glyma19g41480 | EamA-like transporter family | Membrane | - | - | - |  |  |
| Glyma19g41490 | Importin beta binding domain; HEAT repeat; Armadillo/beta-catenin-like repeat | - | Importin alpha-related | Karyopherin (importin) alpha | - |  |  |
| Glyma19g41500 | Plant neutral invertase | Beta-fructofuranosidase | - | - | - |  |  |
| Glyma19g41510 | PsbP | Calcium ion binding; oxygen evolving complex; photosynthesis; extrinsic to membrane | - | - | - |  |  |
| Glyma19g41520 | Adaptor complexes medium subunit family | Vesicle-mediated transport; clathrin adaptor complex; intracellular protein transport | Clathrin coat assembly protein | Clathrin-associated protein medium chain | - |  |  |
| Glyma19g41530 | Eukaryotic protein of unknown function (DUF846) | Integral to membrane | Family not named | - | - |  |  |
| Glyma19g41540.2 | NAD dependent epimerase/dehydratase family | Catalytic activity binding coenzyme binding | NAD dependent epimerase/dehydratase | dTDP-glucose 4-6-dehydratase/UDP-glucuronic acid decarboxylase | Gma.17726.1.S1_at | - | -3 |
| Glyma19g41550.1 | Glycosyl transferase family 8 | Transferase activity, transferring hexosyl groups transferase activity, transferring glycosyl groups | Glycogenin | Glycosyl transferase, family 8 - glycogenin | GmaAffx.80465.1.S1_at | 2 | 2 |
| Glyma19g41560.1 | EamA-like transporter family | - | - | - | Gma.17939.1.S1_at | - | - |
| Glyma19g41580 | Unknown | - | - | - | - |  |  |
| Glyma19g41590.1 | Haloacid dehalogenase-like hydrolase;Redoxin;NHL repeat | Hydrolase activity | 2-deoxyglucose-6-phosphate phosphatase 2 | Predicted haloacid-halidohydrolase and related hydrolases | Gma.14131.1.S1_at | - | - |
|  |  |  |  |  | GmaAffx.26456.1.S1_at | - | - |
|  |  |  |  |  | GmaAffx.76884.1.S1_at | - | - |
|  |  |  |  |  | GmaAffx.83041.1.S1_at | No data | No data |
| Glyma19g41600 | Kelch motif | Protein binding | Kelch repeat domain | Uncharacterized conserved protein, contains kelch repeat | - |  |  |
| Glyma19g41610 | ELK domain; KNOX1 domain; KNOX2 domain | DNA binding | Homeobox protein | Transcription factor MEIS1 and related HOX domain proteins | - |  |  |
| Glyma19g41630.1 | Nicotianamine synthase protein | Nicotianamine synthase activity | - | - | GmaAffx.82770.1.S1_at | -3,-5 | -3,-5 |
| Glyma19g41640 | Unknown | - | - | - | - |  |  |
| Glyma19g41650.1 | PCRF domain|Peptidyl-tRNA hydrolase domain | Translation release factor activity | Peptide chain release factor | - | GmaAffx.42592.1.A1_at | - | 5 |
| Glyma19g41660.2 | Universal stress protein family | Molecular function | - | - | GmaAffx.92672.1.S1_x_at | - | - |
| Glyma19g41660.3 | Universal stress protein family | Molecular function | - | - | GmaAffx.92672.1.S1_s_at | - | 3 |
| Glyma19g41670.1 | ADP-ribosylation factor family|GTPase of unknown function|Miro-like protein | GTP binding | ARF-related | Vesicle coat complex COPII, GTPase subunit SAR1 | Gma.2547.1.S1_at | -2 | -2,5 |
| Glyma19g41680.1 | U1 zinc finger | Nucleic acid binding zinc ion binding | - | U1-like Zn-finger protein | Gma.8365.3.S1_at | -2 | -2 |
| Glyma19g41690 | Thioredoxin; Endoplasmic reticulum protein ERp29, C-terminal domain | Endoplasmic reticulum | Protein disulfide isomerase | Thioredoxin/protein disulfide isomerase | - |  |  |
| Glyma19g41700.1 | Protein of unknown function (DUF1645) | Molecular function | - | - | Gma.10024.1.S1_at | - | - |
|  |  |  |  |  | GmaAffx.16109.1.S1_at | - | -2 |
|  |  |  |  |  | GmaAffx.16109.1.S1_x_at | -2 | 1 |
| Glyma19g41710.1 | - | Molecular function | - | - | GmaAffx.41182.1.S1_at | No data | No data |
| Glyma19g41730.1 | Calmodulin binding protein-like | Calmodulin binding | - | - | GmaAffx.83548.1.S1_at | 2,5 | 2,5 |
| Glyma19g41740.1 | Calmodulin binding protein-like | Calmodulin binding | - | - | GmaAffx.87219.1.S1_at | No data | No data |
| Glyma19g41760.3 | DNAJ domain | Heat shock protein binding | DNAJ/HSP40 | - | Gma.1547.2.S1_a_at | - | - |
| Glyma19g41770.1 | Importin beta binding domain|Armadillo/beta-catenin-like repeat|HEAT repeat | Protein transporter activity | Importin alpha-related | Karyopherin (importin) alpha | GmaAffx.12107.1.S1_at | -2,3,5 | -2,3,5 |
|  |  |  |  |  | GmaAffx.46888.1.A1_at | -2,3 | -2,5 |
| Glyma19g41780 | GATA zinc finger | Transcription factor activity; regulation of transcription, DNA-dependent; zinc ion binding; sequence-specific DNA binding | Transcription factor GATA (GATA binding factor) | - | - |  |  |
| Glyma19g41790 | Exonuclease | - | Subfamily not named | - | - |  |  |
| Glyma19g41800.1 | Kinesin motor domain | ATPase activity microtubule binding microtubule motor activity | Kinesin heavy chain | Kinesin (KAR3 subfamily) | GmaAffx.67321.1.S1_at | - | - |
| Glyma19g41810.2 | Protein phosphatase 2C | Protein serine/threonine phosphatase activity | Protein phosphatase 2c | Serine/threonine protein phosphatase | Gma.15241.1.S1_at | -2,5 | -2,5 |
|  |  |  |  |  | GmaAffx.43743.1.S1_at | -2 | -2 |
| Glyma19g41820.1 | Unknown | Kinase activity | - | - | Gma.5735.1.S1_at | - | -1,-2 |
|  |  |  | - | - | GmaAffx.71360.1.S1_at | - | -2,3 |
| Glyma19g41830 | Glycosyl hydrolases family 16 | Hydrolase activity, hydrolyzing O-glycosyl compounds; carbohydrate metabolism | - | - | - |  |  |
| Glyma19g41840 | LisH; WD domain, G-beta repeat | - | WD40 repeat protein | WD40-repeat-containing subunit of the 18S rRNA processing complex | - |  |  |
| Glyma19g41870.1 | Protein phosphatase 2C | Protein serine/threonine phosphatase activity | Protein phosphatase 2c | Serine/threonine protein phosphatase | Gma.3865.1.S1_at | -2 | -2 |
| Glyma19g41880.2 | - | Molecular function | - | - | GmaAffx.38475.1.S1_at | - | - |
| Glyma19g41890 | Sodium/hydrogen exchanger family | Integral to membrane; solute:hydrogen antiporter activity; cation transport | - | Predicted K+/H+-antiporter | - |  |  |
| Glyma19g41900 | F-box domain | - | - | - | - |  |  |
| Glyma19g41910.1 | Unknown | - | - | - | GmaAffx.74666.1.S1_at | No data | No data |
| Glyma19g41920.1 | Unknown | - | - | - | GmaAffx.47104.1.A1_at | No data | No data |
| Glyma19g41930 | Leucine Rich Repeat | Protein binding | F-box/leucine rich repeat protein | Leucine rich repeat proteins, some proteins contain F-box | - |  |  |
| Glyma19g41950.1 | Plant invertase/pectin methylesterase inhibitor|Pectinesterase | Pectinesterase activity | - | - | GmaAffx.43774.1.A1_at | -2,-3 | -2,-3,-5 |
| Glyma19g41960 | Pectinesterase; Plant invertase/pectin methylesterase inhibitor | Pectinesterase activity; cell wall; cell wall modification | - | - | - |  |  |
| Glyma19g41970 | Pectinesterase; Plant invertase/pectin methylesterase inhibitor | Pectinesterase activity; cell wall; cell wall modification | - | - | - |  |  |
| Glyma19g41980.3 | Unknown | - | - | - | Gma.5988.2.S1_at | - | - |
|  |  |  | - | - | Gma.5988.3.S1_a_at | - | - |
| Glyma19g41990.1 | Alpha/beta hydrolase fold | Epoxide hydrolase activity | Alpha/beta hydrolase related | Soluble epoxide hydrolase | Gma.8022.2.S1_at | No data | No data |
|  |  |  |  |  | GmaAffx.81027.1.A1_at | No data | No data |
| Glyma19g42000.2 | - | Epoxide hydrolase activity | Alpha/beta hydrolase related | - | Gma.6715.1.A1_at | 2,3,5 | 2,3,5 |
|  |  |  |  |  | GmaAffx.93429.2.S1_s_at | 2,3,5 | 2,3,5 |
|  |  |  |  |  | GmaAffx.93429.2.S1_x_at | 2,3,5 | 2,3,5 |
| Glyma19g42030 | Eukaryotic ribosomal protein L18 | - | 60s ribosomal protein l18 | 60s ribosomal protein L18 | - |  |  |
| Glyma19g42040 | MtN3/saliva family | Membrane | Stromal cell protein/nodulin mtn3-related | Multitransmembrane protein | - |  |  |
| Glyma19g42050.1 | Calcineurin-like phosphoesterase | Protein serine/threonine phosphatase activity | Serine/threonine protein phosphatase | Serine/threonine specific protein phosphatase PP1, catalytic subunit | GmaAffx.17486.1.S1_at | 2 | - |
|  |  |  |  |  | GmaAffx.17486.1.S1_s_at | 2 | - |
| Glyma19g42060.2 | Dormancy/auxin associated protein | Molecular function | - | - | Gma.15235.1.S1_at | -2,-3 | -2,-3,-5 |
| Glyma19g42070 | BRCA1 C Terminus (BRCT) domain; Pescadillo N-terminus | Intracellular | Pescadillo - related | Protein required for normal rRNA processing | - |  |  |
| Glyma19g42090.1 | Ribosomal L22e protein family | Structural constituent of ribosome | 60s ribosomal protein l22 | 60S ribosomal protein L22 | GmaAffx.92770.1.S1_at | - | 5 |
| Glyma19g42100 | Zinc finger, C3HC4 type (RING finger) | - | Ring finger protein 24-related | - | - |  |  |
| Glyma19g42110 | ATPase family associated with various cellular activities (AAA) | ATP binding | 26s protease regulatory subunit | 26S proteasome regulatory complex, ATPase RPT5 | - |  |  |
| Glyma19g42120.2 | - | Molecular function | - | Uncharacterized conserved protein | Gma.14232.1.S1_at | -2,-3 | -2,-3,-5 |
| Glyma19g42130.1 | Unknown | Molecular function | - | - | Gma.14077.1.A1_at | - | -3 |
|  |  |  |  |  | Gma.15163.1.S1_a_at | -2,-5 | -2,-3,-5 |
| Glyma19g42140.1 | - | Binding | - | - | GmaAffx.16291.1.S1_at | No data | No data |
| Glyma19g42150.1 | F-actin capping protein alpha subunit | Actin binding | F-actin capping protein alpha | F-actin capping protein, alpha subunit | Gma.1932.1.S1_at | -2 | -2 |
|  |  |  |  |  | Gma.1932.1.S1_s_at | -2,-3 | -2 |
| Glyma19g42160.1 | Acyltransferase | Acyltransferase activity | Taz protein (tafazzin) | Phosphate acyltransferase | GmaAffx.40855.2.S1_at | - | - |
|  |  |  |  |  | GmaAffx.40855.3.S1_at | 2 | - |
|  |  |  |  |  | GmaAffx.40855.4.S1_at | No data | No data |
| Glyma19g42170.1 | Unknown | - | - | - | GmaAffx.75082.1.A1_at | No data | No data |
| Glyma19g42170.2 | Unknown | - | - | - | GmaAffx.51553.1.S1_at | - | - |
| Glyma19g42180.2 | Clp protease | Proteolysis and peptidolysis; serine-type endopeptidase activity | Protease family s14 clpp protease | ATP-dependent Clp protease, proteolytic subunit | Gma.10918.1.S1_at | - | - |
|  |  |  |  |  | GmaAffx.61778.1.S1_at | - | - |
|  |  |  |  |  | GmaAffx.92956.1.S1_s_at | -5 | -3,-5 |
| Glyma19g42200.1 | Rapid ALkalinization Factor (RALF) | Signal transducer activity | - | - | Gma.9498.1.S1_a_at | 2 | - |
| Glyma19g42210.1 | RAD9 | DNA repair | DNA repair protein RAD9 | Checkpoint 9-1-1 complex, RAD9 component | GmaAffx.57323.1.S1_at | No data | No data |
|  |  |  |  |  | GmaAffx.69813.1.A1_at | - | - |
| Glyma19g42220.1 | Respiratory burst NADPH oxidase|EF hand|Ferric reductase like transmembrane component|FAD-binding domain|Ferric reductase NAD binding domain | Calcium ion binding iron ion binding oxidoreductase activity FAD binding | NADPH oxidase | Ferric reductase, NADH/NADPH oxidase and related proteins | GmaAffx.33386.1.A1_at | 2 | 2 |
| Glyma19g42230 | Formin Homology 2 Domain | - | Formin-related | Rho GTPase effector BNI1 and related formins | - |  |  |
| Glyma19g42240.1 | Core histone H2A/H2B/H3/H4|Histone-like transcription factor (CBF/NF-Y) and archaeal histone | DNA binding | Histone H2A | Histone 2A | Gma.13144.1.S1_at | -2,5 | 3,5 |
| Glyma19g42250.2 | Trypsin | Serine-type peptidase activity | Serine protease family s1c htra-related | Serine protease | GmaAffx.50303.1.S1_at | -2,5 | -2 |
|  |  |  |  |  | GmaAffx.50303.2.S1_at | -2,5 | - |
| Glyma19g42260.1 | PWWP domain | Molecular function | - | - | GmaAffx.87428.1.S1_at | No data | No data |
| Glyma19g42270 | Kelch motif | - | Kelch repeat domain | Kelch repeat-containing proteins | - |  |  |
| Glyma19g42280 | Zinc finger, C2H2 type | Intracellular; zinc ion binding | Zinc finger protein | Ovo and related transcription factors | - |  |  |
| Glyma19g42290.1 | CBS domain pair|CBS domain pair | Protein kinase activator activity | Amp-activated protein kinase, gamma regulatory subunit | 5'-AMP-activated protein kinase, gamma subunit | GmaAffx.77312.1.S1_at | - | 5 |
| Glyma19g42310 | Zn-finger in Ran binding protein and others | Zinc ion binding; intracellular | RNA binding protein | - | - |  |  |
| Glyma19g42320.1 | Putative lysophospholipase | Molecular function | Alpha/beta hydrolase related | - | Gma.4389.1.S1_at | -2 | -2 |
| Glyma19g42330.1 | Metallopeptidase family M24; Creatinase/Prolidase N-terminal domain | - | Protease family m24 (methionyl aminopeptidase, aminopeptidase p) | Xaa-Pro aminopeptidase | Gma.16537.1.A1_at | -2 | -2 |
|  |  |  |  |  | Gma.16537.2.S1_at | -2 | - |
|  |  |  |  |  | GmaAffx.36985.1.S1_at | - | - |
|  |  |  |  |  | GmaAffx.56494.1.S1_at | No data | No data |
| Glyma19g42340 | Protein tyrosine kinase | Protein-tyrosine kinase activity; protein amino acid phosphorylation; ATP binding | MAPKK-related serine/threonine protein kinases | MEKK and related serine/threonine protein kinases | - |  |  |
| Glyma19g42350 | Unknown | - | - | - | - |  |  |
| Glyma19g42360.1 | Kinesin motor domain | Microtubule motor activity | Kinesin heavy chain | Kinesin (KAR3 subfamily) | GmaAffx.70296.1.S1_at | - | - |
| Glyma19g42370 | Glycosyl hydrolases family 2, immunoglobulin-like beta-sandwich domain | Carbohydrate metabolism; hydrolase activity, hydrolyzing O-glycosyl compounds | Glycoside hydrolase | - | - |  |  |
| Glyma19g42380 | Glycosyl transferase family 8 | Transferase activity, transferring glycosyl groups | Glycogenin | Glycosyl transferase, family 8 - glycogenin | - |  |  |
| Glyma19g42390 | Cyclin, N-terminal domain | - | Family not named | Cyclin | - |  |  |
| Glyma19g42400.3 | Rrp15p | Molecular function | - | - | Gma.9300.1.S1_at | -2 | - |
|  |  |  |  |  | GmaAffx.77111.1.S1_at | -2 | 5 |
|  |  |  |  |  | GmaAffx.93266.1.S1_s_at | - | - |
| Glyma19g42410.1 | Yip1 domain | Molecular function | Uncharacterized | Uncharacterized conserved protein | Gma.739.1.S1_at | -2 | -2 |
|  |  |  |  |  | Gma.739.2.A1_at | -2 | -2,5 |
| Glyma19g42420 | unknown | - | - | - | - |  |  |
| Glyma19g42440.1 | GHMP kinases N terminal domain|GHMP kinases C terminal | Mevalonate kinase activity | Mevalonate kinase/galactokinase | Mevalonate kinase MVK/ERG12 | GmaAffx.86817.1.S1_at | -2 | -2 |
| Glyma19g42450 | PPR repeat | - | Pentatricopeptide repeat-containing protein | - | - |  |  |
| Glyma19g42460.1 | Core histone H2A/H2B/H3/H4|Histone-like transcription factor (CBF/NF-Y) and archaeal histone | DNA binding transcription factor activity | Histone-like transcription factor ccaat-related | CCAAT-binding factor, subunit C (HAP5) | GmaAffx.86595.1.S1_at | -2 | -2 |
| Glyma19g42480.1 | Signal peptidase subunit | Signal peptide processing | Microsomal signal peptidase 23 kd subunit (spc22/23) | Signal peptidase complex subunit | Gma.12215.1.S1_at | -2,3,5 | -2,3 |
| Glyma19g42490 | - | - | Aspartyl proteases | Aspartyl protease | - |  |  |
| Glyma19g42500 | - | - | Copper chaperone-related | - | - |  |  |
| Glyma19g42510.1 | Zinc finger, C3HC4 type (RING finger) | Protein binding zinc ion binding | Ring finger and protease associated domain-containing | - | GmaAffx.90914.1.S1_at | 2 | - |
| Glyma19g42520 | Helix-loop-helix DNA-binding domain | Transcription regulator activity; regulation of transcription | - | - | - |  |  |
| Glyma19g42530.1 | Aminotransferase class IV | Catalytic activity | Subgroup iiii aminotransferase | Branched chain aminotransferase BCAT1, pyridoxal phosphate enzymes type IV superfamily | Gma.1702.1.A1_at | No data | No data |
| Glyma19g42540.2 | Putative methyltransferase | Molecular function | Uncharacterized | Predicted methyltransferase | Gma.4015.1.S1_at | -2 | - |
| Glyma19g42560.1 | GDSL-like Lipase/Acylhydrolase | Carboxylesterase activity | Zinc finger fyve domain containing protein | - | GmaAffx.11201.1.A1_at | -2,-3 | -2,-3 |
| Glyma19g42580 | Kinesin motor domain | Microtubule-based movement; microtubule motor activity; ATP binding | Kinesin heavy chain | - | - |  |  |
| Glyma19g42590 | Unknown | - | - | - | - |  |  |
| Glyma19g42600.1 | Eukaryotic glutathione synthase, ATP binding domain|Eukaryotic glutathione synthase | Glutathione synthase activity | Glutathione synthetase | Glutathione synthetase | Gma.3133.1.S1_at | - | -2 |
| Glyma19g42600.2 | Eukaryotic glutathione synthase, ATP binding domain|Eukaryotic glutathione synthase | glutathione synthase activity | Glutathione synthetase | Glutathione synthetase | GmaAffx.71453.1.S1_at | -2 | 1 |
| Glyma19g42610 | Eukaryotic glutathione synthase | Glutathione synthase activity; ATP binding | Glutathione synthetase | Glutathione synthetase | - |  |  |
| Glyma19g42620 | Eukaryotic glutathione synthase | Glutathione synthase activity; ATP binding | Glutathione synthetase | Glutathione synthetase | - |  |  |
| Glyma19g42630.1 | Protein of unknown function (DUF1068) | Molecular function | - | - | Gma.12538.1.A1_at | -2,-3 | -2,-3 |
|  |  |  |  |  | GmaAffx.45862.1.S1_at | -2 | -2 |
|  |  |  |  |  | GmaAffx.45862.1.S1_s_at | -2 | -2,-3 |
| Glyma19g42640 | Phosphoglycerate mutase family | - | Phosphoglycerate mutase | Phosphoglycerate mutase | - |  |  |
| Glyma19g42660 | - | - | Uncharacterized | Uncharacterized conserved protein | - |  |  |
| Glyma19g42670 | Protein of unknown function (DUF3595) | - | Uncharacterized | - | - |  |  |
| Glyma19g42680 | Protein of unknown function (DUF3049) | - | - | - | - |  |  |
| Glyma19g42690 | Major Facilitator Superfamily | Transmembrane transport | Sugar transporter | Predicted transporter (major facilitator superfamily) | - |  |  |
| Glyma19g42700.1 | Ribosomal protein S11 | Protein biosynthesis | 40s ribosomal protein s14/30s ribosomal protein s11 | 40S ribosomal protein S14 | Gma.15476.1.S1_at | - | -2 |
|  |  |  |  |  | Gma.15476.1.S1_x_at | - | - |
| Glyma19g42710 | - | - | Sugar transporter | Predicted transporter (major facilitator superfamily) | - |  |  |

| ^a^: PFAM description provided by the Soybean Genome Project, DoE Joint Genome Institute ( <http://www.phytozome.net/soybean.php>, updated on July 2011); |
| --- |
| ^b^: Gene Ontology Descriptions obtained from the ([http://soybase.org](http://soybase.org/), updated on July 2011); |
| ^c^: PANTHER description provided by the Soybean Genome Project, DoE Joint Genome Institute (<http://www.phytozome.net/soybean.php>, updated on July 2011); |
| ^d^: KOG Description assigned by the Soybean Genome Project, DoE Joint Genome Institute (http://www.phytozome.net/soybean.php, updated on July 2011);  ^e^: Affymetrix probe IDs that match with the predicted genes underlying QTL (<http://soybase.org/AffyChip/>), where - means there was no Affymetrix IDs available for the specific gene;  ^f^: IR: Infection response—significant differences in transcript abundance of inoculated samples compared to mock-inoculated samples in Conrad (C) or Sloan (S) (microarray data, TST-FDR, *P* < 0.05) at specified time points (dai); where - means there was no significant response observed at any time point after inoculation, a positive value indicates that the gene was up-regulated at the specified time point, and a negative value indicates the gene was down-regulated at the specified time point. |
