# Supplementary material for: Dissection of two soybean QTL conferring partial resistance to Phytophthora sojae through sequence and gene expression analysis
Source: BMC Genomics. 2012 Aug 28;13:428. doi: 10.1186/1471-2164-13-428 (PMC3443417; doi:10.1186/1471-2164-13-428)
Supplement: Additional file 3 — SNPs detected between Conrad and Sloan in sequenced genes underlying QTL 19–1 and 19–2. [file 1471-2164-13-428-S3.docx]

Additional file 3. SNPs detected between Conrad and Sloan in sequenced genes underlying QTL 19-1 and 19-2.

| **Gene** | **Nucleotides**^a^ | | **SNP coordinates in Williams 82** |
| --- | --- | --- | --- |
|  | **Conrad** | **Sloan** |  |
| Glyma19g35270 | T | A | 42812032 |
| Glyma19g35270 | C | A | 42812476 |
| Glyma19g35270 | T | C | 42812483 |
| Glyma19g35270 | A | C | 42812535 |
| Glyma19g35270 | G | A | 42812555 |
| Glyma19g35270 | A | C | 42812750 |
| Glyma19g35270 | T | C | 42812863 |
| Glyma19g35270 | A | T | 42813115 |
| Glyma19g35270 | C | A | 42813785 |
| Glyma19g35270 | G | A | 42814148 |
| Glyma19g35270 | G | C | 42814753 |
| Glyma19g35270 | C | A | 42815622 |
| Glyma19g35270 | T | A | 42815985 |
| Glyma19g35290 | G | A | 42831152 |
| Glyma19g35320 | C | T | 42860961 |
| Glyma19g35330 | G | T | 42868487 |
| Glyma19g35330 | A | G | 42868736 |
| Glyma19g35330 | A | G | 42868915 |
| Glyma19g35330 | A | C | 42869047 |
| Glyma19g35330 | T | G | 42869235 |
| Glyma19g35330 | A | T | 42869406 |
| Glyma19g35330 | G | A | 42871377 |
| Glyma19g35330 | G | A | 42872344 |
| Glyma19g35330 | A | G | 42873873 |
| Glyma19g35330 | A | G | 42874304 |
| Glyma19g35330 | T | G | 42875058 |
| Glyma19g35330 | A | C | 42875139 |
| Glyma19g35330 | A | G | 42875185 |
| Glyma19g35330 | T | C | 42875471 |
| Glyma19g35330 | A | G | 42875788 |
| Glyma19g35340 | G | T | 42878485 |
| Glyma19g35340 | T | C | 42878589 |
| Glyma19g35340 | G | C | 42878598 |
| Glyma19g35340 | A | T | 42878702 |
| Glyma19g35340 | A | G | 42879074 |
| Glyma19g35340 | A | G | 42879650 |
| Glyma19g35340 | C | A | 42880267 |
| Glyma19g35340 | A | G | 42881531 |
| Glyma19g35340 | A | G | 42881656 |
| Glyma19g35340 | T | C | 42881808 |
| Glyma19g35340 | C | T | 42882352 |
| Glyma19g35340 | A | G | 42882403 |
| Glyma19g35340 | A | G | 42882405 |
| Glyma19g35340 | T | A | 42882418 |
| Glyma19g35340 | G | T | 42882437 |
| Glyma19g35340 | A | C | 42882516 |
| Glyma19g35340 | C | T | 42882662 |
| Glyma19g35340 | C | A | 42882708 |
| Glyma19g35340 | A | T | 42882957 |
| Glyma19g35360 | C | G | 42894479 |
| Glyma19g35360 | G | A | 42894501 |
| Glyma19g35370 | G | A | 42898134 |
| Glyma19g35370 | T | A | 42898830 |
| Glyma19g35370 | C | T | 42898858 |
| Glyma19g35370 | A | G | 42898885 |
| Glyma19g35370 | G | A | 42898895 |
| Glyma19g35370 | A | G | 42899024 |
| Glyma19g35370 | G | A | 42899113 |
| Glyma19g35370 | A | G | 42899133 |
| Glyma19g35370 | C | A | 42899137 |
| Glyma19g35370 | G | A | 42899139 |
| Glyma19g35370 | C | T | 42899154 |
| Glyma19g35370 | G | T | 42899241 |
| Glyma19g35370 | A | T | 42899250 |
| Glyma19g35370 | C | A | 42899292 |
| Glyma19g35370 | A | G | 42899309 |
| Glyma19g35370 | T | C | 42899467 |
| Glyma19g35370 | T | C | 42899520 |
| Glyma19g35370 | G | A | 42899554 |
| Glyma19g35370 | C | T | 42899596 |
| Glyma19g35370 | C | A | 42899804 |
| Glyma19g35370 | C | A | 42899862 |
| Glyma19g35370 | A | C | 42899895 |
| Glyma19g35370 | A | G | 42899897 |
| Glyma19g35370 | A | G | 42899901 |
| Glyma19g35370 | G | A | 42900108 |
| Glyma19g35370 | G | T | 42900171 |
| Glyma19g35370 | A | G | 42900190 |
| Glyma19g35370 | A | G | 42900287 |
| Glyma19g35370 | G | A | 42900418 |
| Glyma19g35370 | C | T | 42900861 |
| Glyma19g35370 | A | C | 42900971 |
| Glyma19g35370 | G | A | 42901181 |
| Glyma19g35370 | C | T | 42901415 |
| Glyma19g35410 | G | C | 42941182 |
| Glyma19g35450 | G | T | 43028426 |
| Glyma19g35450 | A | T | 43028507 |
| Glyma19g35450 | G | T | 43028824 |
| Glyma19g35450 | T | A | 43028831 |
| Glyma19g35450 | G | A | 43028853 |
| Glyma19g35450 | G | A | 43029755 |
| Glyma19g35450 | C | T | 43030013 |
| Glyma19g35450 | A | G | 43030068 |
| Glyma19g35450 | A | C | 43030122 |
| Glyma19g35450 | C | G | 43030292 |
| Glyma19g35450 | A | T | 43030821 |
| Glyma19g35450 | T | A | 43031216 |
| Glyma19g35450 | A | G | 43031441 |
| Glyma19g35450 | T | C | 43031537 |
| Glyma19g35450 | C | A | 43032506 |
| Glyma19g35450 | A | T | 43032523 |
| Glyma19g35450 | G | A | 43032692 |
| Glyma19g35450 | G | T | 43032878 |
| Glyma19g35450 | A | G | 43033272 |
| Glyma19g35450 | C | G | 43034048 |
| Glyma19g35450 | T | G | 43034408 |
| Glyma19g35450 | G | C | 43034698 |
| Glyma19g35450 | A | G | 43035398 |
| Glyma19g35450 | A | T | 43035562 |
| Glyma19g35450 | C | A | 43035705 |
| Glyma19g35450 | C | T | 43035949 |
| Glyma19g35450 | G | T | 43036067 |
| Glyma19g35450 | T | A | 43036146 |
| Glyma19g35450 | C | A | 43036191 |
| Glyma19g35450 | C | A | 43036373 |
| Glyma19g35450 | C | T | 43036384 |
| Glyma19g35450 | T | C | 43036392 |
| Glyma19g35450 | T | C | 43036429 |
| Glyma19g35450 | G | A | 43036434 |
| Glyma19g35450 | G | A | 43036468 |
| Glyma19g40800 | T | G | 47113470 |
| Glyma19g40800 | C | A | 47113475 |
| Glyma19g40800 | G | A | 47113847 |
| Glyma19g40800 | G | A | 47113947 |
| Glyma19g40800 | A | C | 47114076 |
| Glyma19g40800 | C | T | 47114162 |
| Glyma19g40800 | A | T | 47114182 |
| Glyma19g40800 | G | A | 47114303 |
| Glyma19g40800 | A | T | 47114388 |
| Glyma19g40800 | G | T | 47114567 |
| Glyma19g40800 | T | C | 47114806 |
| Glyma19g40800 | C | T | 47115078 |
| Glyma19g40800 | T | A | 47115085 |
| Glyma19g40800 | A | G | 47115100 |
| Glyma19g40800 | G | A | 47115202 |
| Glyma19g40800 | T | G | 47115439 |
| Glyma19g40800 | C | T | 47115461 |
| Glyma19g40800 | T | G | 47115572 |
| Glyma19g40800 | C | T | 47115581 |
| Glyma19g40800 | C | A | 47115662 |
| Glyma19g40800 | C | G | 47115853 |
| Glyma19g40800 | C | G | 47116418 |
| Glyma19g40800 | A | G | 47116517 |
| Glyma19g40800 | A | G | 47116586 |
| Glyma19g40800 | G | A | 47116637 |
| Glyma19g40800 | T | C | 47116821 |
| Glyma19g40800 | C | T | 47116928 |
| Glyma19g40800 | G | T | 47116951 |
| Glyma19g40800 | C | T | 47117018 |
| Glyma19g40800 | C | A | 47117166 |
| Glyma19g40800 | A | G | 47117173 |
| Glyma19g40800 | T | G | 47117211 |
| Glyma19g40800 | G | A | 47117364 |
| Glyma19g40800 | T | G | 47117541 |
| Glyma19g40800 | C | T | 47117545 |
| Glyma19g40800 | T | A | 47117630 |
| Glyma19g40800 | G | T | 47117655 |
| Glyma19g40800 | T | A | 47117920 |
| Glyma19g40800 | G | A | 47118102 |
| Glyma19g40800 | A | G | 47118156 |
| Glyma19g40800 | C | T | 47118234 |
| Glyma19g40800 | T | C | 47118485 |
| Glyma19g40800 | A | C | 47118697 |
| Glyma19g40800 | G | A | 47119034 |
| Glyma19g40800 | A | G | 47119179 |
| Glyma19g40800 | C | T | 47119212 |
| Glyma19g40800 | G | T | 47119323 |
| Glyma19g40840 | C | G | 47154294 |
| Glyma19g40940 | A | T | 47220633 |
| Glyma19g40940 | A | G | 47221974 |
| Glyma19g40980 | G | A | 47277652 |
| Glyma19g41020 | C | T | 47336187 |
| Glyma19g41020 | C | T | 47336198 |
| Glyma19g41020 | T | G | 47336349 |
| Glyma19g41020 | G | A | 47336514 |
| Glyma19g41020 | T | A | 47336753 |
| Glyma19g41020 | T | A | 47336939 |
| Glyma19g41020 | G | A | 47337241 |
| Glyma19g41020 | T | G | 47338437 |
| Glyma19g41030 | T | C | 47341872 |
| Glyma19g41030 | C | G | 47341906 |
| Glyma19g41030 | G | A | 47342635 |
| Glyma19g41030 | A | T | 47343796 |
| Glyma19g41030 | C | A | 47344920 |
| Glyma19g41030 | T | A | 47345389 |
| Glyma19g41030 | C | T | 47345743 |
| Glyma19g41030 | A | G | 47347173 |
| Glyma19g41030 | A | T | 47347321 |
| Glyma19g41030 | A | C | 47347365 |
| Glyma19g41030 | C | T | 47347559 |
| Glyma19g41030 | T | C | 47347798 |
| Glyma19g41030 | G | A | 47347956 |
| Glyma19g41030 | T | C | 47348048 |
| Glyma19g41030 | A | G | 47348144 |
| Glyma19g41030 | A | G | 47348210 |
| Glyma19g41030 | G | A | 47348416 |
| Glyma19g41030 | T | A | 47348504 |
| Glyma19g41030 | G | A | 47348630 |
| Glyma19g41070 | A | C | 47389099 |
| Glyma19g41070 | A | G | 47390304 |
| Glyma19g41080 | G | A | 47392861 |
| Glyma19g41080 | C | G | 47393322 |
| Glyma19g41080 | T | C | 47393325 |
| Glyma19g41080 | A | G | 47394320 |
| Glyma19g41100 | A | T | 47413113 |
| Glyma19g41100 | A | T | 47413118 |
| Glyma19g41100 | G | A | 47413205 |
| Glyma19g41100 | T | C | 47413883 |
| Glyma19g41100 | C | T | 47415021 |
| Glyma19g41100 | T | G | 47415425 |
| Glyma19g41100 | A | G | 47415825 |
| Glyma19g41100 | G | A | 47416130 |
| Glyma19g41100 | T | C | 47416682 |
| Glyma19g41100 | T | A | 47417069 |
| Glyma19g41100 | C | A | 47417084 |
| Glyma19g41100 | G | A | 47417475 |
| Glyma19g41110 | G | A | 47426256 |
| Glyma19g41110 | A | T | 47426285 |
| Glyma19g41110 | A | T | 47426292 |
| Glyma19g41110 | H | A | 47427537 |
| Glyma19g41110 | H | T | 47427541 |
| Glyma19g41110 | T | C | 47427637 |
| Glyma19g41110 | T | G | 47432687 |
| Glyma19g41110 | G | A | 47432869 |
| Glyma19g41110 | A | G | 47432896 |
| Glyma19g41110 | A | G | 47432926 |
| Glyma19g41110 | G | A | 47432934 |
| Glyma19g41110 | T | C | 47432935 |
| Glyma19g41110 | T | A | 47432948 |
| Glyma19g41120 | G | A | 47438503 |
| Glyma19g41120 | G | A | 47440708 |
| Glyma19g41120 | G | A | 47441404 |
| Glyma19g41120 | A | G | 47442634 |
| Glyma19g41120 | A | C | 47442735 |
| Glyma19g41120 | T | A | 47442791 |
| Glyma19g41120 | A | C | 47442947 |
| Glyma19g41120 | C | A | 47443110 |
| Glyma19g41120 | T | C | 47443279 |
| Glyma19g41120 | A | G | 47443511 |
| Glyma19g41120 | T | A | 47444279 |
| Glyma19g41180 | A | G | 47487812 |
| Glyma19g41180 | G | A | 47487985 |
| Glyma19g41180 | T | A | 47488001 |
| Glyma19g41180 | C | T | 47488323 |
| Glyma19g41180 | C | T | 47488329 |
| Glyma19g41180 | A | G | 47488470 |
| Glyma19g41210 | T | A | 47512367 |
| Glyma19g41210 | A | G | 47512633 |
| Glyma19g41210 | T | C | 47513062 |
| Glyma19g41210 | G | A | 47514601 |
| Glyma19g41210 | G | T | 47514644 |
| Glyma19g41230 | H | T | 47533400 |
| Glyma19g41230 | T | A | 47533679 |
| Glyma19g41230 | A | G | 47534386 |
| Glyma19g41230 | T | C | 47534864 |
| Glyma19g41230 | C | T | 47534984 |
| Glyma19g41230 | C | A | 47535046 |
| Glyma19g41230 | A | T | 47535447 |
| Glyma19g41230 | G | A | 47535485 |
| Glyma19g41230 | A | C | 47535644 |
| Glyma19g41230 | T | C | 47535996 |
| Glyma19g41230 | A | T | 47536079 |
| Glyma19g41230 | A | C | 47536082 |
| Glyma19g41230 | C | G | 47536102 |
| Glyma19g41230 | C | G | 47536159 |
| Glyma19g41230 | A | G | 47536571 |
| Glyma19g41240 | A | C | 47542210 |
| Glyma19g41240 | A | G | 47542213 |
| Glyma19g41240 | A | T | 47542254 |
| Glyma19g41240 | C | A | 47542260 |
| Glyma19g41240 | A | G | 47542462 |
| Glyma19g41240 | C | T | 47542577 |
| Glyma19g41240 | A | T | 47542788 |
| Glyma19g41240 | A | C | 47544766 |
| Glyma19g41240 | C | T | 47544809 |
| Glyma19g41240 | C | T | 47545859 |
| Glyma19g41240 | C | T | 47545947 |
| Glyma19g41240 | C | A | 47547064 |
| Glyma19g41250 | A | G | 47558283 |
| Glyma19g41260 | G | T | 47572115 |
| Glyma19g41260 | C | A | 47572643 |
| Glyma19g41260 | A | G | 47573277 |
| Glyma19g41260 | A | C | 47573490 |
| Glyma19g41260 | A | T | 47573708 |
| Glyma19g41390 | C | T | 47665927 |
| Glyma19g41390 | A | T | 47666094 |
| Glyma19g41390 | G | T | 47668434 |
| Glyma19g41390 | A | T | 47668686 |
| Glyma19g41390 | T | A | 47668702 |
| Glyma19g41390 | T | A | 47668707 |
| Glyma19g41390 | T | G | 47668717 |
| Glyma19g41390 | A | G | 47668801 |
| Glyma19g41390 | T | C | 47668826 |
| Glyma19g41390 | G | A | 47668842 |
| Glyma19g41390 | G | A | 47668923 |
| Glyma19g41390 | T | G | 47668941 |
| Glyma19g41390 | T | C | 47669220 |
| Glyma19g41390 | T | C | 47669307 |
| Glyma19g41400 | T | G | 47672158 |
| Glyma19g41400 | A | G | 47672198 |
| Glyma19g41400 | A | T | 47672289 |
| Glyma19g41400 | G | A | 47672300 |
| Glyma19g41400 | C | G | 47672331 |
| Glyma19g41400 | G | A | 47672492 |
| Glyma19g41400 | T | G | 47672720 |
| Glyma19g41400 | C | G | 47672799 |
| Glyma19g41400 | G | A | 47672837 |
| Glyma19g41400 | A | G | 47672914 |
| Glyma19g41400 | A | G | 47675207 |
| Glyma19g41400 | C | A | 47675379 |
| Glyma19g41400 | C | T | 47675775 |
| Glyma19g41400 | T | A | 47675846 |
| Glyma19g41400 | C | T | 47675889 |
| Glyma19g41400 | G | A | 47675968 |
| Glyma19g41400 | A | C | 47676038 |
| Glyma19g41400 | T | C | 47676504 |
| Glyma19g41400 | C | A | 47679017 |
| Glyma19g41400 | A | G | 47680025 |
| Glyma19g41400 | C | G | 47680274 |
| Glyma19g41400 | A | G | 47681104 |
| Glyma19g41400 | C | T | 47681235 |
| Glyma19g41400 | T | C | 47681354 |
| Glyma19g41400 | G | A | 47681933 |
| Glyma19g41400 | T | G | 47682550 |
| Glyma19g41410 | T | G | 47687357 |
| Glyma19g41410 | T | A | 47688158 |
| Glyma19g41410 | T | A | 47688159 |
| Glyma19g41410 | T | C | 47688206 |
| Glyma19g41410 | A | T | 47688752 |
| Glyma19g41410 | T | C | 47688770 |
| Glyma19g41410 | C | T | 47688899 |
| Glyma19g41410 | A | G | 47688901 |
| Glyma19g41410 | C | T | 47688904 |
| Glyma19g41410 | C | T | 47688910 |
| Glyma19g41410 | G | A | 47688924 |
| Glyma19g41410 | G | A | 47688927 |
| Glyma19g41410 | T | C | 47688947 |
| Glyma19g41410 | C | t | 47688954 |
| Glyma19g41410 | T | C | 47688955 |
| Glyma19g41410 | T | C | 47689042 |
| Glyma19g41410 | G | A | 47689146 |
| Glyma19g41410 | C | T | 47690423 |
| Glyma19g41410 | A | T | 47690724 |
| Glyma19g41410 | T | A | 47690935 |
| Glyma19g41420 | A | T | 47694376 |
| Glyma19g41420 | A | G | 47694420 |
| Glyma19g41420 | C | A | 47694423 |
| Glyma19g41420 | C | A | 47694425 |
| Glyma19g41420 | T | A | 47694427 |
| Glyma19g41420 | T | G | 47694516 |
| Glyma19g41420 | T | A | 47694595 |
| Glyma19g41420 | G | T | 47694734 |
| Glyma19g41420 | G | A | 47694772 |
| Glyma19g41420 | G | A | 47694776 |
| Glyma19g41420 | G | A | 47694795 |
| Glyma19g41420 | A | G | 47694863 |
| Glyma19g41420 | T | A | 47695050 |
| Glyma19g41420 | T | C | 47695061 |
| Glyma19g41420 | T | C | 47695095 |
| Glyma19g41420 | T | A | 47695180 |
| Glyma19g41420 | T | A | 47695425 |
| Glyma19g41420 | G | A | 47695567 |
| Glyma19g41420 | C | A | 47697604 |
| Glyma19g41420 | C | T | 47698080 |
| Glyma19g41420 | T | C | 47698817 |
| Glyma19g41420 | T | A | 47699657 |
| Glyma19g41420 | C | A | 47699660 |
| Glyma19g41420 | T | G | 47699849 |
| Glyma19g41420 | G | A | 47699995 |
| Glyma19g41440 | T | C | 47707851 |
| Glyma19g41440 | C | G | 47710070 |
| Glyma19g41440 | T | A | 47711777 |
| Glyma19g41440 | T | A | 47712182 |
| Glyma19g41520 | C | A | 47771747 |
| Glyma19g41520 | T | G | 47773448 |
| Glyma19g41580 | G | C | 47808583 |
| Glyma19g41580 | T | A | 47809240 |
| Glyma19g41590 | C | T | 47825055 |
| Glyma19g41600 | G | A | 47841925 |
| Glyma19g41600 | A | C | 47843643 |
| Glyma19g41600 | T | C | 47843867 |
| Glyma19g41630 | C | G | 47867018 |
| Glyma19g41630 | C | T | 47867334 |
| Glyma19g41630 | G | A | 47867381 |
| Glyma19g41630 | T | C | 47868132 |
| Glyma19g41650 | G | T | 47881321 |
| Glyma19g41650 | T | C | 47881355 |
| Glyma19g41650 | G | A | 47881524 |
| Glyma19g41650 | G | A | 47881841 |
| Glyma19g41650 | T | G | 47882655 |
| Glyma19g41650 | A | C | 47882710 |
| Glyma19g41650 | T | A | 47882747 |
| Glyma19g41650 | A | G | 47882778 |
| Glyma19g41650 | T | G | 47882784 |
| Glyma19g41650 | A | G | 47882796 |
| Glyma19g41650 | C | T | 47882893 |
| Glyma19g41650 | C | T | 47882931 |
| Glyma19g41650 | A | G | 47882969 |
| Glyma19g41650 | T | C | 47882985 |
| Glyma19g41680 | C | T | 47893105 |
| Glyma19g41680 | T | A | 47893170 |
| Glyma19g41680 | A | G | 47893499 |
| Glyma19g41680 | G | A | 47893620 |
| Glyma19g41680 | A | G | 47893768 |
| Glyma19g41680 | C | G | 47893904 |
| Glyma19g41680 | G | A | 47893961 |
| Glyma19g41680 | A | T | 47893997 |
| Glyma19g41680 | A | C | 47894485 |
| Glyma19g41680 | A | G | 47894550 |
| Glyma19g41680 | T | A | 47894638 |
| Glyma19g41680 | A | C | 47894652 |
| Glyma19g41680 | A | G | 47894670 |
| Glyma19g41680 | G | A | 47894683 |
| Glyma19g41680 | C | A | 47894713 |
| Glyma19g41680 | A | T | 47894790 |
| Glyma19g41680 | A | C | 47894819 |
| Glyma19g41680 | G | A | 47894846 |
| Glyma19g41680 | T | C | 47895366 |
| Glyma19g41680 | T | A | 47895797 |
| Glyma19g41680 | A | T | 47895798 |
| Glyma19g41680 | G | A | 47895837 |
| Glyma19g41680 | A | T | 47896049 |
| Glyma19g41680 | G | A | 47896058 |
| Glyma19g41680 | T | C | 47896312 |
| Glyma19g41680 | A | T | 47896490 |
| Glyma19g41680 | G | A | 47896537 |
| Glyma19g41680 | G | A | 47896548 |
| Glyma19g41680 | A | T | 47896727 |
| Glyma19g41680 | A | T | 47896728 |
| Glyma19g41690 | T | C | 47899440 |
| Glyma19g41690 | G | A | 47899531 |
| Glyma19g41690 | A | G | 47899680 |
| Glyma19g41690 | G | A | 47899990 |
| Glyma19g41690 | A | T | 47900088 |
| Glyma19g41690 | G | A | 47900688 |
| Glyma19g41690 | A | G | 47900974 |
| Glyma19g41690 | T | C | 47901067 |
| Glyma19g41690 | T | C | 47901152 |
| Glyma19g41690 | G | A | 47901470 |
| Glyma19g41690 | T | A | 47901595 |
| Glyma19g41690 | T | G | 47901734 |
| Glyma19g41690 | G | T | 47901735 |
| Glyma19g41690 | G | A | 47902229 |
| Glyma19g41690 | C | G | 47902470 |
| Glyma19g41690 | A | G | 47902477 |
| Glyma19g41690 | A | C | 47902765 |
| Glyma19g41700 | C | A | 47905843 |
| Glyma19g41700 | C | T | 47906358 |
| Glyma19g41700 | C | A | 47906429 |
| Glyma19g41700 | T | C | 47907038 |
| Glyma19g41700 | A | T | 47907866 |
| Glyma19g41700 | T | C | 47907903 |
| Glyma19g41700 | T | A | 47908163 |
| Glyma19g41700 | A | G | 47908347 |
| Glyma19g41700 | G | A | 47908448 |
| Glyma19g41700 | T | A | 47908865 |
| Glyma19g41740 | t | A | 47936502 |
| Glyma19g41740 | t | A | 47936504 |
| Glyma19g41740 | H | A | 47936506 |
| Glyma19g41740 | A | T | 47937131 |
| Glyma19g41740 | G | A | 47938156 |
| Glyma19g41740 | G | A | 47938538 |
| Glyma19g41740 | C | T | 47938844 |
| Glyma19g41740 | A | G | 47938853 |
| Glyma19g41740 | C | A | 47939444 |
| Glyma19g41740 | T | G | 47939823 |
| Glyma19g41740 | C | T | 47939969 |
| Glyma19g41740 | C | T | 47940003 |
| Glyma19g41740 | G | A | 47940515 |
| Glyma19g41740 | C | A | 47940855 |
| Glyma19g41740 | T | A | 47941225 |
| Glyma19g41780 | G | T | 47958361 |
| Glyma19g41780 | A | C | 47958362 |
| Glyma19g41780 | A | C | 47959245 |
| Glyma19g41790 | G | T | 47960638 |
| Glyma19g41790 | A | T | 47960920 |
| Glyma19g41790 | G | A | 47960940 |
| Glyma19g41790 | G | A | 47960984 |
| Glyma19g41790 | T | A | 47961359 |
| Glyma19g41790 | C | T | 47961838 |
| Glyma19g41790 | G | C | 47961906 |
| Glyma19g41790 | G | T | 47962098 |
| Glyma19g41790 | T | A | 47962385 |
| Glyma19g41790 | G | T | 47962504 |
| Glyma19g41790 | C | T | 47962578 |
| Glyma19g41790 | C | T | 47962611 |
| Glyma19g41790 | C | A | 47962875 |
| Glyma19g41790 | G | A | 47962972 |
| Glyma19g41790 | C | G | 47963052 |
| Glyma19g41790 | G | A | 47963405 |
| Glyma19g41790 | A | G | 47963458 |
| Glyma19g41790 | G | A | 47963465 |
| Glyma19g41790 | G | C | 47964066 |
| Glyma19g41790 | G | A | 47964297 |
| Glyma19g41790 | A | G | 47964300 |
| Glyma19g41790 | T | G | 47964440 |
| Glyma19g41790 | T | G | 47964587 |
| Glyma19g41790 | T | A | 47964588 |
| Glyma19g41790 | C | G | 47964592 |
| Glyma19g41790 | C | T | 47965418 |
| Glyma19g41790 | G | A | 47965975 |
| Glyma19g41790 | A | G | 47966077 |
| Glyma19g41800 | T | A | 47972272 |
| Glyma19g41800 | T | A | 47972436 |
| Glyma19g41800 | A | T | 47972515 |
| Glyma19g41800 | C | T | 47972547 |
| Glyma19g41800 | A | T | 47972644 |
| Glyma19g41800 | C | T | 47972870 |
| Glyma19g41800 | A | G | 47973103 |
| Glyma19g41800 | G | T | 47973243 |
| Glyma19g41800 | C | T | 47973526 |
| Glyma19g41800 | A | C | 47973527 |
| Glyma19g41800 | G | A | 47973629 |
| Glyma19g41800 | G | A | 47973684 |
| Glyma19g41800 | A | G | 47974006 |
| Glyma19g41800 | C | A | 47974078 |
| Glyma19g41800 | A | G | 47974098 |
| Glyma19g41800 | T | A | 47974179 |
| Glyma19g41800 | C | G | 47974243 |
| Glyma19g41800 | G | C | 47974369 |
| Glyma19g41800 | G | A | 47974428 |
| Glyma19g41800 | G | A | 47974765 |
| Glyma19g41800 | G | A | 47974997 |
| Glyma19g41800 | A | T | 47975000 |
| Glyma19g41800 | T | C | 47975072 |
| Glyma19g41800 | T | C | 47975132 |
| Glyma19g41800 | G | A | 47975403 |
| Glyma19g41800 | T | C | 47975521 |
| Glyma19g41800 | A | T | 47976772 |
| Glyma19g41800 | G | A | 47976774 |
| Glyma19g41800 | G | T | 47976858 |
| Glyma19g41800 | G | A | 47976916 |
| Glyma19g41800 | C | T | 47977498 |
| Glyma19g41800 | G | A | 47977689 |
| Glyma19g41820 | A | T | 47992220 |
| Glyma19g41820 | T | A | 47993714 |
| Glyma19g41820 | T | A | 47993743 |
| Glyma19g41820 | G | A | 47994044 |
| Glyma19g41820 | T | C | 47994289 |
| Glyma19g41830 | A | T | 47998201 |
| Glyma19g41830 | A | T | 47998305 |
| Glyma19g41830 | A | G | 47998952 |
| Glyma19g41830 | H | C | 47999761 |
| Glyma19g41840 | G | A | 48000021 |
| Glyma19g41840 | T | G | 48002266 |
| Glyma19g41840 | C | T | 48002371 |
| Glyma19g41840 | A | T | 48002849 |
| Glyma19g41840 | T | C | 48004698 |
| Glyma19g41840 | T | A | 48005407 |
| Glyma19g41870 | T | A | 48018210 |
| Glyma19g41870 | A | T | 48019265 |
| Glyma19g41870 | C | A | 48021012 |
| Glyma19g41870 | T | G | 48021065 |
| Glyma19g41870 | T | C | 48021428 |
| Glyma19g41870 | C | T | 48021632 |
| Glyma19g41870 | T | C | 48022081 |
| Glyma19g41880 | T | C | 48029758 |
| Glyma19g41880 | G | A | 48029764 |
| Glyma19g41880 | A | G | 48029789 |
| Glyma19g41880 | T | C | 48029829 |
| Glyma19g41880 | A | G | 48029836 |
| Glyma19g41880 | T | C | 48029847 |
| Glyma19g41880 | C | G | 48029959 |
| Glyma19g41880 | A | C | 48030062 |
| Glyma19g41880 | A | G | 48030205 |
| Glyma19g41880 | C | T | 48030678 |
| Glyma19g41880 | C | A | 48030803 |
| Glyma19g41880 | T | C | 48030946 |
| Glyma19g41880 | A | G | 48031432 |
| Glyma19g41880 | A | G | 48031993 |
| Glyma19g41880 | T | C | 48032123 |
| Glyma19g41880 | G | T | 48032235 |
| Glyma19g41880 | C | A | 48032242 |
| Glyma19g41880 | T | C | 48032548 |
| Glyma19g41880 | G | A | 48032627 |
| Glyma19g41880 | G | A | 48032836 |
| Glyma19g41880 | G | A | 48032877 |
| Glyma19g41880 | C | T | 48032980 |
| Glyma19g41880 | T | C | 48033109 |
| Glyma19g41880 | T | C | 48033123 |
| Glyma19g41880 | C | A | 48033231 |
| Glyma19g41880 | G | A | 48033498 |
| Glyma19g41880 | T | C | 48033499 |
| Glyma19g41880 | A | T | 48033848 |
| Glyma19g41880 | A | C | 48034140 |
| Glyma19g41880 | G | A | 48034269 |
| Glyma19g41880 | C | T | 48034478 |
| Glyma19g41880 | A | T | 48034487 |
| Glyma19g41880 | G | C | 48034488 |
| Glyma19g41880 | T | C | 48034528 |
| Glyma19g41880 | T | A | 48035513 |
| Glyma19g41880 | G | A | 48035943 |
| Glyma19g41880 | T | G | 48036043 |
| Glyma19g41880 | T | G | 48036114 |
| Glyma19g41880 | C | A | 48036163 |
| Glyma19g41880 | T | C | 48036192 |
| Glyma19g41880 | C | T | 48036283 |
| Glyma19g41880 | C | T | 48036365 |
| Glyma19g41880 | C | T | 48036442 |
| Glyma19g41880 | C | T | 48036475 |
| Glyma19g41880 | T | C | 48036562 |
| Glyma19g41890 | A | C | 48038306 |
| Glyma19g41890 | A | G | 48038710 |
| Glyma19g41890 | G | T | 48038831 |
| Glyma19g41890 | T | C | 48041399 |
| Glyma19g41900 | C | G | 48048691 |
| Glyma19g41900 | C | T | 48048732 |
| Glyma19g41900 | C | T | 48049092 |
| Glyma19g41900 | C | T | 48049237 |
| Glyma19g41900 | G | A | 48049382 |
| Glyma19g41900 | A | C | 48049439 |
| Glyma19g41900 | C | T | 48050493 |
| Glyma19g41900 | A | T | 48050590 |
| Glyma19g42070 | A | T | 48149413 |
| Glyma19g42070 | A | C | 48149423 |
| Glyma19g42120 | G | A | 48182461 |
| Glyma19g42160 | A | G | 48200568 |
| Glyma19g42160 | T | A | 48201172 |
| Glyma19g42160 | G | C | 48202330 |
| Glyma19g42160 | A | C | 48202887 |
| Glyma19g42170 | A | T | 48206053 |
| Glyma19g42170 | G | A | 48206064 |
| Glyma19g42170 | G | T | 48206943 |
| Glyma19g42180 | C | T | 48208447 |
| Glyma19g42180 | G | A | 48208513 |
| Glyma19g42180 | C | G | 48208656 |
| Glyma19g42180 | A | C | 48209100 |
| Glyma19g42180 | G | A | 48209165 |
| Glyma19g42180 | G | C | 48209615 |
| Glyma19g42180 | G | A | 48209643 |
| Glyma19g42180 | T | G | 48209644 |
| Glyma19g42180 | C | A | 48209823 |
| Glyma19g42180 | A | C | 48209960 |
| Glyma19g42180 | A | G | 48210185 |
| Glyma19g42180 | C | T | 48210187 |
| Glyma19g42180 | T | G | 48210245 |
| Glyma19g42180 | T | C | 48210389 |
| Glyma19g42180 | G | A | 48210391 |
| Glyma19g42180 | C | T | 48210576 |
| Glyma19g42180 | G | A | 48210599 |
| Glyma19g42180 | H | G | 48210731 |
| Glyma19g42180 | H | T | 48210738 |
| Glyma19g42180 | H | T | 48210740 |
| Glyma19g42180 | H | A | 48210741 |
| Glyma19g42180 | H | A | 48211421 |
| Glyma19g42180 | A | G | 48211999 |
| Glyma19g42180 | C | T | 48213230 |
| Glyma19g42180 | C | A | 48213347 |
| Glyma19g42180 | C | G | 48213558 |
| Glyma19g42180 | T | A | 48213601 |
| Glyma19g42180 | A | T | 48213607 |
| Glyma19g42180 | A | C | 48213884 |
| Glyma19g42200 | C | T | 48220487 |
| Glyma19g42200 | C | T | 48220500 |
| Glyma19g42200 | A | T | 48220605 |
| Glyma19g42200 | A | G | 48220666 |
| Glyma19g42200 | A | C | 48220772 |
| Glyma19g42200 | A | T | 48220793 |
| Glyma19g42200 | C | A | 48220847 |
| Glyma19g42200 | G | C | 48220905 |
| Glyma19g42200 | A | T | 48220949 |
| Glyma19g42200 | C | T | 48221270 |
| Glyma19g42200 | G | A | 48221473 |
| Glyma19g42210 | G | A | 48232086 |
| Glyma19g42210 | T | G | 48233704 |
| Glyma19g42210 | T | C | 48234932 |
| Glyma19g42210 | A | G | 48235046 |
| Glyma19g42210 | G | A | 48235067 |
| Glyma19g42210 | C | A | 48235125 |
| Glyma19g42210 | C | A | 48235171 |
| Glyma19g42210 | T | C | 48235244 |
| Glyma19g42210 | A | T | 48235388 |
| Glyma19g42210 | T | C | 48235446 |
| Glyma19g42210 | A | T | 48235581 |
| Glyma19g42210 | G | C | 48235593 |
| Glyma19g42220 | T | A | 48238950 |
| Glyma19g42220 | C | T | 48238955 |
| Glyma19g42220 | C | G | 48239628 |
| Glyma19g42220 | A | G | 48240340 |
| Glyma19g42220 | T | A | 48240654 |
| Glyma19g42220 | G | A | 48240969 |
| Glyma19g42220 | C | T | 48241345 |
| Glyma19g42220 | A | G | 48243402 |
| Glyma19g42220 | G | C | 48243483 |
| Glyma19g42220 | C | T | 48243485 |
| Glyma19g42220 | G | C | 48243491 |
| Glyma19g42220 | A | T | 48243492 |
| Glyma19g42220 | T | G | 48243497 |
| Glyma19g42220 | C | G | 48243505 |
| Glyma19g42220 | A | T | 48243536 |
| Glyma19g42220 | C | G | 48243554 |
| Glyma19g42220 | A | G | 48243563 |
| Glyma19g42220 | T | A | 48243720 |
| Glyma19g42220 | G | A | 48243790 |
| Glyma19g42220 | G | A | 48244253 |
| Glyma19g42220 | G | A | 48244529 |
| Glyma19g42220 | t | A | 48244596 |
| Glyma19g42220 | T | A | 48244597 |
| Glyma19g42220 | H | C | 48244650 |
| Glyma19g42220 | C | T | 48245363 |
| Glyma19g42220 | T | C | 48245888 |
| Glyma19g42220 | G | T | 48245934 |
| Glyma19g42220 | G | A | 48246140 |
| Glyma19g42220 | T | A | 48246310 |
| Glyma19g42240 | A | T | 48260513 |
| Glyma19g42240 | T | C | 48260980 |
| Glyma19g42330 | A | G | 48348307 |
| Glyma19g42330 | C | T | 48348661 |
| Glyma19g42330 | G | C | 48349477 |
| Glyma19g42330 | A | G | 48349514 |
| Glyma19g42330 | T | C | 48349565 |
| Glyma19g42330 | C | T | 48349718 |
| Glyma19g42330 | T | C | 48349929 |
| Glyma19g42330 | G | A | 48350099 |
| Glyma19g42330 | T | G | 48350902 |
| Glyma19g42330 | T | C | 48351009 |
| Glyma19g42330 | T | A | 48351571 |
| Glyma19g42330 | T | C | 48351635 |
| Glyma19g42330 | T | C | 48351759 |
| Glyma19g42330 | G | A | 48351944 |
| Glyma19g42330 | G | A | 48351996 |
| Glyma19g42330 | T | A | 48352514 |
| Glyma19g42330 | T | C | 48352657 |
| Glyma19g42330 | T | C | 48353473 |
| Glyma19g42330 | T | A | 48353687 |
| Glyma19g42330 | T | A | 48353974 |
| Glyma19g42330 | C | T | 48354026 |
| Glyma19g42330 | T | C | 48354264 |
| Glyma19g42330 | A | T | 48354297 |
| Glyma19g42330 | T | G | 48354468 |
| Glyma19g42330 | G | A | 48354523 |
| Glyma19g42330 | A | G | 48354588 |
| Glyma19g42330 | G | A | 48354617 |
| Glyma19g42330 | C | T | 48354747 |
| Glyma19g42330 | A | G | 48354763 |
| Glyma19g42330 | T | C | 48354853 |
| Glyma19g42330 | A | G | 48355087 |
| Glyma19g42330 | A | G | 48355246 |
| Glyma19g42330 | A | C | 48355438 |
| Glyma19g42340 | G | T | 48356932 |
| Glyma19g42340 | G | A | 48356964 |
| Glyma19g42340 | C | A | 48357098 |
| Glyma19g42340 | T | A | 48357109 |
| Glyma19g42340 | C | T | 48357147 |
| Glyma19g42340 | A | T | 48357322 |
| Glyma19g42340 | A | G | 48357586 |
| Glyma19g42340 | C | T | 48360615 |
| Glyma19g42340 | A | G | 48360634 |
| Glyma19g42340 | T | A | 48360708 |
| Glyma19g42340 | T | C | 48362328 |
| Glyma19g42360 | C | t | 48370716 |
| Glyma19g42360 | T | A | 48370741 |
| Glyma19g42360 | A | H | 48370783 |
| Glyma19g42360 | A | t | 48370806 |
| Glyma19g42360 | A | H | 48370898 |
| Glyma19g42360 | A | t | 48370917 |
| Glyma19g42360 | G | A | 48370922 |
| Glyma19g42360 | T | C | 48370925 |
| Glyma19g42360 | G | A | 48370926 |
| Glyma19g42360 | C | t | 48370946 |
| Glyma19g42360 | A | g | 48371148 |
| Glyma19g42360 | T | A | 48371151 |
| Glyma19g42360 | T | A | 48371172 |
| Glyma19g42360 | T | C | 48371175 |
| Glyma19g42360 | A | H | 48371180 |
| Glyma19g42360 | C | H | 48371181 |
| Glyma19g42360 | A | G | 48371999 |
| Glyma19g42360 | T | C | 48372586 |
| Glyma19g42360 | C | T | 48372726 |
| Glyma19g42360 | T | C | 48373747 |
| Glyma19g42360 | C | T | 48374511 |
| Glyma19g42370 | A | G | 48378286 |
| Glyma19g42390 | C | A | 48391250 |
| Glyma19g42390 | T | C | 48391469 |
| Glyma19g42390 | A | T | 48391474 |
| Glyma19g42390 | C | A | 48391490 |
| Glyma19g42390 | T | A | 48391503 |
| Glyma19g42390 | A | T | 48392499 |
| Glyma19g42390 | C | A | 48392549 |
| Glyma19g42390 | A | G | 48392998 |
| Glyma19g42420 | C | A | 48406672 |
| Glyma19g42420 | G | A | 48406674 |
| Glyma19g42420 | T | C | 48406869 |
| Glyma19g42420 | A | G | 48406908 |
| Glyma19g42420 | C | G | 48407061 |
| Glyma19g42420 | A | G | 48407722 |
| Glyma19g42420 | T | C | 48408139 |
| Glyma19g42420 | A | G | 48408269 |
| Glyma19g42420 | C | T | 48408722 |
| Glyma19g42420 | A | T | 48408856 |
| Glyma19g42420 | C | T | 48408980 |
| Glyma19g42420 | A | T | 48409017 |
| Glyma19g42420 | T | A | 48409121 |
| Glyma19g42440 | G | A | 48410402 |
| Glyma19g42440 | G | A | 48410404 |
| Glyma19g42440 | G | A | 48410406 |
| Glyma19g42440 | G | A | 48410408 |
| Glyma19g42440 | A | T | 48410415 |
| Glyma19g42440 | C | T | 48410417 |
| Glyma19g42440 | A | G | 48410444 |
| Glyma19g42440 | A | G | 48410446 |
| Glyma19g42440 | A | G | 48410448 |
| Glyma19g42440 | A | G | 48410450 |
| Glyma19g42440 | T | A | 48412431 |
| Glyma19g42440 | T | G | 48412549 |
| Glyma19g42440 | A | T | 48412698 |
| Glyma19g42440 | G | T | 48413806 |
| Glyma19g42440 | A | c | 48413807 |
| Glyma19g42450 | T | G | 48414200 |
| Glyma19g42450 | G | A | 48414240 |
| Glyma19g42450 | A | T | 48414473 |
| Glyma19g42450 | A | G | 48415462 |
| Glyma19g42450 | T | C | 48415811 |
| Glyma19g42450 | T | C | 48415870 |
| Glyma19g42450 | C | T | 48415971 |
| Glyma19g42460 | A | T | 48419045 |
| Glyma19g42460 | A | T | 48419936 |
| Glyma19g42460 | G | A | 48421449 |
| Glyma19g42460 | A | G | 48421517 |
| Glyma19g42480 | T | C | 48428897 |
| Glyma19g42480 | A | T | 48429916 |
| Glyma19g42480 | G | A | 48430980 |
| Glyma19g42480 | A | G | 48430992 |
| Glyma19g42480 | C | T | 48431042 |
| Glyma19g42480 | G | T | 48431151 |
| Glyma19g42480 | G | A | 48431586 |
| Glyma19g42480 | G | C | 48431645 |
| Glyma19g42480 | T | A | 48431867 |
| Glyma19g42490 | G | A | 48436356 |
| Glyma19g42490 | A | G | 48436686 |
| Glyma19g42490 | G | A | 48437087 |
| Glyma19g42490 | G | T | 48437134 |
| Glyma19g42490 | G | A | 48437163 |
| Glyma19g42490 | G | A | 48437189 |
| Glyma19g42490 | T | A | 48437193 |
| Glyma19g42490 | A | T | 48437204 |
| Glyma19g42490 | A | C | 48437223 |
| Glyma19g42500 | T | C | 48443205 |
| Glyma19g42500 | A | G | 48444369 |
| Glyma19g42500 | A | T | 48444617 |
| Glyma19g42510 | G | C | 48457222 |
| Glyma19g42510 | A | C | 48457900 |
| Glyma19g42510 | A | G | 48457939 |
| Glyma19g42510 | C | T | 48458238 |
| Glyma19g42510 | C | H | 48458246 |
| Glyma19g42510 | G | A | 48458291 |
| Glyma19g42510 | C | G | 48458389 |
| Glyma19g42510 | T | C | 48458599 |
| Glyma19g42510 | G | A | 48458610 |
| Glyma19g42510 | G | C | 48458615 |
| Glyma19g42510 | T | G | 48458726 |
| Glyma19g42510 | C | T | 48458728 |
| Glyma19g42510 | G | C | 48458792 |
| Glyma19g42510 | G | C | 48458879 |
| Glyma19g42510 | A | C | 48458924 |
| Glyma19g42510 | A | G | 48458969 |
| Glyma19g42510 | T | C | 48459017 |
| Glyma19g42520 | C | T | 48476141 |
| Glyma19g42520 | C | G | 48476391 |
| Glyma19g42520 | T | A | 48476405 |
| Glyma19g42520 | T | C | 48476453 |
| Glyma19g42520 | T | A | 48476487 |
| Glyma19g42520 | G | A | 48476802 |
| Glyma19g42520 | A | G | 48477367 |
| Glyma19g42520 | A | T | 48477871 |
| Glyma19g42520 | C | G | 48479120 |
| Glyma19g42530 | C | T | 48492605 |
| Glyma19g42530 | C | A | 48493980 |
| Glyma19g42530 | C | T | 48495433 |
| Glyma19g42530 | C | T | 48495683 |
| Glyma19g42560 | T | A | 48509656 |
| Glyma19g42560 | C | T | 48510461 |
| Glyma19g42560 | G | C | 48511111 |
| Glyma19g42560 | T | A | 48511265 |
| Glyma19g42560 | G | A | 48511455 |
| Glyma19g42560 | A | G | 48511689 |
| Glyma19g42560 | T | C | 48512233 |
| Glyma19g42560 | G | A | 48512539 |
| Glyma19g42560 | C | A | 48512571 |
| Glyma19g42560 | G | A | 48512572 |
| Glyma19g42590 | T | A | 48519554 |
| Glyma19g42590 | A | G | 48521375 |
| Glyma19g42600 | A | C | 48522540 |
| Glyma19g42600 | T | C | 48522881 |
| Glyma19g42600 | A | G | 48522991 |
| Glyma19g42600 | T | C | 48523012 |
| Glyma19g42600 | A | G | 48523079 |
| Glyma19g42600 | A | G | 48523139 |
| Glyma19g42600 | T | C | 48523155 |
| Glyma19g42600 | G | A | 48523178 |
| Glyma19g42600 | G | T | 48523258 |
| Glyma19g42600 | C | G | 48523281 |
| Glyma19g42600 | G | A | 48523370 |
| Glyma19g42600 | G | C | 48523745 |
| Glyma19g42600 | G | A | 48525742 |
| Glyma19g42600 | A | G | 48526188 |
| Glyma19g42600 | C | A | 48526228 |
| Glyma19g42600 | G | A | 48526265 |
| Glyma19g42600 | A | C | 48526480 |
| Glyma19g42600 | T | C | 48526481 |
| Glyma19g42600 | A | G | 48526950 |
| Glyma19g42600 | C | T | 48526990 |
| Glyma19g42600 | T | C | 48527082 |
| Glyma19g42600 | T | A | 48527094 |
| Glyma19g42600 | C | T | 48527101 |
| Glyma19g42600 | T | A | 48527604 |
| Glyma19g42600 | G | T | 48527713 |
| Glyma19g42600 | T | A | 48527929 |
| Glyma19g42600 | C | G | 48528187 |
| Glyma19g42600 | A | G | 48528303 |
| Glyma19g42600 | G | A | 48528461 |
| Glyma19g42600 | C | G | 48528485 |
| Glyma19g42600 | T | C | 48528552 |
| Glyma19g42600 | T | A | 48528794 |
| Glyma19g42600 | T | C | 48528990 |
| Glyma19g42600 | G | C | 48529242 |
| Glyma19g42600 | C | T | 48529260 |
| Glyma19g42600 | C | T | 48529296 |
| Glyma19g42600 | A | G | 48529380 |
| Glyma19g42600 | G | A | 48529673 |
| Glyma19g42600 | T | C | 48529680 |
| Glyma19g42600 | T | C | 48529720 |
| Glyma19g42610 | C | T | 48537961 |
| Glyma19g42610 | A | T | 48538335 |
| Glyma19g42610 | T | A | 48538380 |
| Glyma19g42610 | C | H | 48538384 |
| Glyma19g42610 | G | A | 48538386 |
| Glyma19g42610 | T | C | 48538387 |
| Glyma19g42610 | T | A | 48538389 |
| Glyma19g42610 | T | A | 48538588 |
| Glyma19g42610 | C | A | 48538590 |
| Glyma19g42610 | G | A | 48538610 |
| Glyma19g42610 | C | A | 48538874 |
| Glyma19g42610 | T | C | 48538956 |
| Glyma19g42610 | T | C | 48539026 |
| Glyma19g42610 | A | G | 48539128 |
| Glyma19g42610 | T | G | 48539177 |
| Glyma19g42610 | A | C | 48539387 |
| Glyma19g42610 | A | C | 48539423 |
| Glyma19g42610 | G | T | 48539560 |
| Glyma19g42610 | G | T | 48539568 |
| Glyma19g42610 | A | T | 48539836 |
| Glyma19g42610 | T | A | 48539853 |
| Glyma19g42610 | G | C | 48539963 |
| Glyma19g42610 | G | A | 48540387 |
| Glyma19g42610 | A | G | 48540479 |
| Glyma19g42610 | G | A | 48540686 |
| Glyma19g42610 | A | C | 48540751 |
| Glyma19g42610 | C | T | 48540816 |
| Glyma19g42610 | G | A | 48540990 |
| Glyma19g42610 | G | A | 48541199 |
| Glyma19g42610 | A | G | 48541321 |
| Glyma19g42610 | G | A | 48541387 |
| Glyma19g42610 | G | T | 48541462 |
| Glyma19g42610 | G | C | 48541491 |
| Glyma19g42610 | G | C | 48541506 |
| Glyma19g42610 | A | C | 48541522 |
| Glyma19g42610 | G | A | 48541579 |
| Glyma19g42610 | C | G | 48541823 |
| Glyma19g42610 | A | G | 48541842 |
| Glyma19g42610 | T | G | 48541863 |
| Glyma19g42610 | A | C | 48542200 |
| Glyma19g42610 | A | C | 48542261 |
| Glyma19g42610 | A | T | 48542277 |
| Glyma19g42610 | C | G | 48542365 |
| Glyma19g42630 | G | A | 48551990 |
| Glyma19g42630 | T | A | 48553177 |
| Glyma19g42630 | A | T | 48554178 |
| Glyma19g42630 | A | G | 48554273 |
| Glyma19g42640 | A | G | 48556525 |
| Glyma19g42640 | T | C | 48556609 |
| Glyma19g42640 | G | C | 48557415 |
| Glyma19g42640 | T | C | 48558146 |
| Glyma19g42640 | C | A | 48559073 |
| Glyma19g42640 | G | t | 48559082 |
| Glyma19g42640 | T | A | 48559083 |
| Glyma19g42640 | G | A | 48559088 |
| Glyma19g42640 | T | C | 48559125 |
| Glyma19g42640 | A | C | 48559127 |
| Glyma19g42660 | C | T | 48575459 |
| Glyma19g42660 | C | A | 48579453 |
| Glyma19g42660 | T | C | 48579558 |
| Glyma19g42660 | T | A | 48581383 |
| Glyma19g42660 | A | G | 48581449 |
| Glyma19g42660 | T | C | 48581499 |
| Glyma19g42660 | T | A | 48582455 |
| Glyma19g42670 | A | C | 48586043 |
| Glyma19g42670 | A | G | 48586073 |
| Glyma19g42680 | G | C | 48594642 |
| Glyma19g42680 | A | G | 48594712 |
| Glyma19g42680 | A | T | 48594743 |
| Glyma19g42680 | A | G | 48595356 |
| Glyma19g42680 | A | T | 48595418 |
| Glyma19g42680 | A | G | 48595433 |
| Glyma19g42680 | A | T | 48595551 |
| Glyma19g42690 | G | A | 48596308 |
| Glyma19g42690 | A | T | 48596764 |
| Glyma19g42690 | G | T | 48598282 |
| Glyma19g42690 | C | A | 48598829 |
| Glyma19g42690 | T | G | 48599082 |
| Glyma19g42690 | C | T | 48600671 |
| Glyma19g42700 | C | T | 48601124 |
| Glyma19g42700 | A | G | 48601637 |
| Glyma19g42700 | A | T | 48602553 |
| Glyma19g42700 | T | C | 48602680 |
| Glyma19g42700 | A | C | 48604065 |
| Glyma19g42710 | A | G | 48604270 |
| Glyma19g42710 | A | G | 48606617 |
| Glyma19g42710 | C | T | 48606661 |
| Glyma19g42710 | A | G | 48606739 |
| Glyma19g42710 | A | G | 48606742 |
| Glyma19g42710 | T | C | 48606761 |
| Glyma19g42710 | G | T | 48607161 |
| Glyma19g42710 | G | T | 48607213 |
| Glyma19g42710 | G | A | 48607262 |
| Glyma19g42710 | T | A | 48607263 |
| Glyma19g42710 | G | A | 48607331 |
| Glyma19g42710 | G | A | 48607333 |
| Glyma19g42710 | T | G | 48607335 |
| Glyma19g42710 | A | G | 48607339 |
| Glyma19g42710 | C | G | 48607340 |
| Glyma19g42710 | T | G | 48607342 |
| Glyma19g42710 | T | C | 48607348 |
| Glyma19g42710 | T | A | 48607353 |
| Glyma19g42710 | A | G | 48607713 |

^a^A heterozygous locus is represented by “H”.
