# Supplementary material for: Dissection of two soybean QTL conferring partial resistance to Phytophthora sojae through sequence and gene expression analysis
Source: BMC Genomics. 2012 Aug 28;13:428. doi: 10.1186/1471-2164-13-428 (PMC3443417; doi:10.1186/1471-2164-13-428)
Supplement: Additional file 4 — Sequence polymorphisms in Conrad (C) in comparison to Williams82 (W) and Sloan (S) in Glyma19g41420 and Glyma19g41800, respectively. [file 1471-2164-13-428-S4.docx]

A.

Glyma19g41420_W/S

AACATCACATCAACAAAAAATATTGGTAACATATATCACATGAATATAATTTCATGTAAAAAA

Glyma19g41420_C

AACATCACATCAACAAAAAATATTGGTAACATATATCACA---------------------------------------------T

B.

Glyma19g41800_W/S

MGLPSFEASDLEQGGKSSRIVNCVLALKSHAERKFGGGNGSSKYSGVAKPPTTGKTLLRKNSEPFMKSMWTMPSGDRDGYMSDPGHDLNERGSVSSLNSLVRQYLSDKKPEEIPTVVESLLSKVMEEFEHHMQIRQEMMEEKEDEQDEQDEHDLQDEQNIQDKQEENYEEKYNKREDSSRQILILKQQNIVETQNRSIQVIFNIYKNLILTFIVHQTKLGMQFMQNEHQKEIINLSKHLHSLASAASGYHKVLDENRKLYNIVQDLKGNIRVYCRVRPFLGGQLSHYSSVGNVEEGSISIITPSKYGKEGKKTFNFNRVFGPSATQGEVFADTQPLIRSVLDGYNVCIFAYGQTGSGKTFTMSGPDDINEETIGVNYRALKDLFYLSEQRKDTISYEISVQMLEIYNEQVRDLLTTDEIRNSSHNGINVPDADLVPVSCTSDVINLMNLGQKNRAVGSTAMNDRSSRSHSCLTVHVQGKNLTSGSTIRGSMHLVDLAGSERADKTEATGDRMKEAQHINKSLSALGDVISSLAQKNAHVPYRNSKLTQLLQDSLGGQAKTLMFVHISPEPEALGETLSTLKFAERVSTVELGAARVNKDNSDVKELKEQIASLKAALARKEGGEAEHFQQSANSSSHEIPKLKSYASSPPMQRSLIGGARKLPKDDSSSLNGQKNAASKLKRRSLDLHDMRKNSPPWPPVRSHRKEDDKESISGDWVDKISINRNDSLTSDDSLVGQWETESKQSSPIKDNQELFDMAITDESDELEIATSDSSESDLHWPAHIPKPITVSSGLGIKARKKPINLRPTKSLEARSMIPSLIPIPVPSRKQPTLVTPARKTPGSIDVKRRIGNAK

Glyma19g41800_C

MGLPSFEASDLEQGGKSSRIVNCVLALKSHAERKFGGGNGSSKYSGVAKPPTTGKTLLRKNSEPFMKSMWTMPSGDRDGYMSDPGHDLNERGSVSSLNSLVRQYLSDKKPEEIPTVVESLLSKVMEEFEHHMQIRQEMMEEMEDEQDEQDEHDLQDEQNIQDNQEENYEEKYNKREDSSRQILILKQQNIVETQNRSIQVIFNISTOP

Additional file 4. Sequence polymorphisms in Conrad (C) in comparison to Williams82 (W) and Sloan (S) in Glyma19g41420 and Glyma19g41800, respectively. W/S indicates that W and S share the same sequence. A. Nucleotide sequence comparison in the 979-917 bp upstream (Gm19: 47694364 to 47694427) of Glyma19g41420; B. Predicted peptide sequence comparison of Glyma19g41800.
